# Is exposure to family member incarceration during childhood linked to diabetes in adulthood? Findings from a representative community sample

**DOI:** 10.1177/2050312120905165

**Published:** 2020-02-05

**Authors:** Bradley A White, Keri J West, Esme Fuller-Thomson

**Affiliations:** 1 Center for Youth Development and Intervention, The University of Alabama, Tuscaloosa, AL, USA; 2 Factor-Inwentash Faculty of Social Work, University of Toronto, Toronto, ON, Canada; 3 Institute for Life Course and Aging, University of Toronto, Toronto, ON, Canada; 4 Department of Family and Community Medicine, University of Toronto, Toronto, ON, Canada

**Keywords:** Diabetes, endocrinology, risk factors, gender, adverse childhood experiences, incarceration

## Abstract

**Objectives:**

Diabetes is a prevalent and serious public health problem, particularly among older adults. A robust literature has shown that adverse childhood experiences contribute to the development of health problems in later life, including diabetes. Family member incarceration during childhood is an under-investigated yet increasingly common adverse childhood experience in the United States. The purpose of this study was to investigate the relationship between family member incarceration during childhood and diabetes in adulthood, while considering the role of gender as well as the impact of a range of potential confounds.

**Methods:**

A large representative community sample of adults aged 40 and older (n = 8790 men, 14,255 women) was drawn from the Behavioral Risk Factor Surveillance System 2012 optional adverse childhood experiences module to investigate the association between family member incarceration during childhood and diabetes. For each gender, nine logistic regression analyses were conducted using distinct clusters of variables (e.g. socioeconomic status and health behaviors).

**Results:**

Among males, the odds of diabetes among those exposed to family member incarceration during childhood ranged from 2.00 to 1.59. In the fully adjusted model, they had elevated odds of 1.64 (95% confidence interval = 1.27, 2.11). Among women, the odds of diabetes was much lower, hovering around 1.00.

**Conclusion:**

Findings suggest that family member incarceration during childhood is associated with diabetes in men, even after adjusting for a wide range of potential risk factors (e.g. sociodemographics, health behaviors, healthcare access, and childhood risk factors). Future research should explore the mechanisms linking family member incarceration during childhood and long-term negative health outcomes in men.

## Introduction

Diabetes mellitus is an increasingly common and serious public health problem, particularly among older adults. Approximately one-quarter of American adults over age 65 have diabetes and almost half have prediabetes.^1^ Diabetes is a chronic disease associated with inflammation and metabolic dysfunction and is characterized by chronic hyperglycemia (high blood sugar) secondary to impaired insulin production, secretion, and/or sensitivity.^2^ There are two predominant persistent types of diabetes. Type 1 is far less prevalent, typically develops in childhood or adolescence, and is an idiopathic autoimmune disorder wherein pancreatic beta cells, which produce insulin, are destroyed by one’s own immune system.^2^ The vast majority (approximately 95%) of all cases of diabetes are type 2, which typically onsets in mid to late life and involves a combination of hepatic or peripheral insulin resistance and beta cell dysfunction, contributing to an inability to suppress glucose production, inadequate glucose uptake, and relative insulin deficiency.^2,3^

Diabetes is among the leading causes of death for those over age 65 and projections suggest the prevalence of diabetes in the United States will at least double over the next three decades, with up to one-third of citizens diagnosed with the disease by 2050.^4,5^ Diabetes also represents a substantial economic burden. In 2012, in the United States, the estimated combined direct and indirect costs associated with diagnosed diabetes was approximately US$245 billion annually, which fails to account for the less tangible yet substantial psychosocial burden, including effects on patients’ quality of life and impacts on loved ones.^6^ Given the magnitude of this problem and significant impacts on mortality and morbidity, further research to better understand etiological risk factors for diabetes is warranted.

Whereas sociodemographic, familial, and proximal lifestyle and physical health risk factors for diabetes have been identified (e.g. being older, obese, sedentary, or of a non-European American race or ethnicity increases risk of type 2),^5^ far less is known about distal influences. However, over the past two decades, a body of literature has demonstrated that adverse childhood experiences (ACEs) including neglect, abuse, and household difficulties not only disrupt a child’s experience of stability, safety, and nurturance, but also contribute to the development of various health problems in adulthood.^7–9^ While there has been limited empirical investigation of the relationship between early adversities and diabetes, a recent systematic review and meta-analysis of seven studies totaling over 87,000 participants found that exposure to certain ACEs—specifically, physical and sexual abuse, neglect, and wartime evacuation and separation from parents—increased the risk of developing diabetes later in life by 32% on average, with neglect having the strongest impact and physical abuse the lowest among these ACEs.^10^

Exposure to the incarceration of a family member during one’s childhood is an under-investigated yet increasingly common ACE for children in the United States. The prevalence of incarceration has dramatically increased in recent decades,^11^ and many state and federal inmates are parents of youth under the age of 18.^12^ Although the experience of family member incarceration during childhood (FMIC) may have beneficial aspects in terms of reducing child exposure to parental criminal activity and associated risks, the impact of incarceration itself is disruptive to family stability, including marriages, jobs, and housing.^13^ Improving the understanding of the long-term biopsychosocial and physical health impacts of FMIC can help inform clinical approaches to assessment and intervention with FMIC-exposed individuals, as well as criminal justice reform.

A growing literature supports the link between FMIC and both psychosocial and physical health outcomes. Recent investigations suggest that FMIC predicts a variety of health concerns related to inflammation, such as asthma, elevated cholesterol,^14^ and myocardial infarction.^8^

Early adverse experiences may influence later life health outcomes via interwoven biopsychosocial processes that unfold over the course of development.^7^ Because “health is not a state but a lifetime achievement,”^15^ the life-course approach is a useful theoretical framework for understanding pathophysiology, particularly for diseases with multiple etiological influences that interact over the course of development. The life-course model helps to identify exposures at critical developmental stages that may elevate disease risk in later life and informs early intervention efforts and the development of policies that can improve health trajectories.^16^

Current theories of biological impacts of stressors support the expectation that ACEs would increase the risk of diabetes in later life. In particular, Hertzman’s theory of biological embedding proposes that early adversities can disrupt the development of systems involved in stress and inflammation response (e.g. the hypothalamus–pituitary–adrenal axis), chronically altering the individual’s metabolic and inflammatory responses to stressors (e.g. as indexed by elevated C-reactive protein),^17^ which can in turn lead to organ system dysfunction and pathology.^18^ While the etiology of diabetes is not fully elucidated, emerging literature implicates the contribution of inflammatory processes in insulin resistance and metabolic dysfunction.^19^ Further supporting this perspective, ACEs have been shown to be associated with dysregulated stress responsivity in adulthood, including elevated inflammation,^15^ which in turn appears to contribute to the development of metabolic disorders, including diabetes.^20^ Furthermore, oxidative stress, defined as an imbalance between the production of free radicals (reactive oxygen species) by subcellular components (e.g. mitochondria) and their neutralization by antioxidants, is similarly associated with both early adversity and with the pathogenesis of type 2 diabetes.^21,22^

Despite the prevalence of incarceration in the United States, few studies on ACEs have considered the experience of FMIC as a potential risk factor for physical health problems in later life.^8^ For instance, the aforementioned systematic review of ACEs and diabetes did not report on any studies examining FMIC.^10^ Furthermore, much of the existing research on the role of ACEs in diabetes is based on clinical samples and does not control for a number of potentially confounding variables.^7,23^ One recent study on health effects of parental incarceration in young adults (mean age 28.8 years; n = 15,701) did consider diabetes.^14^ Parental incarceration was common in this sample (12.5%), predominantly of fathers (85%). However, no association was observed between parental incarceration and diabetes, perhaps because diabetes prevalence was quite low (2.6%), although parental incarceration was associated with other more prevalent physical health effects (e.g. elevated cholesterol, asthma, migraines).^14^

There is growing evidence that ACEs may differentially impact long-term health outcomes based on gender, with impacts being stronger for men.^8,24^ Men appear to be especially vulnerable to biological embedding of early adversities and they exhibit more cortisol reactivity to stress than do women.^25,26^ In addition, studies suggest that stress is associated with a reduction in testosterone levels in men and that the cortisol-to-testosterone ratio is associated with insulin resistance syndrome.^27,28^ Furthermore, men are incarcerated in the United States at much higher rates than women, and the majority of these men have children under age 18.^12^ Incarceration interferes with the ability of fathers to maintain contact with children,^29^ and loss of contact with fathers may be particularly impactful on their sons,^30^ interfering with emotion regulation and ability to cope with daily stressors into adulthood. Men who experienced paternal absence in childhood tend to have elevated cortisol levels, in contrast to women who experienced paternal absence.^31^ Finally, girls and women are more likely to pursue psychosocial support following adversities than are boys and men.^32^

### Objectives

Based on the conceptual and empirical foundations discussed above, the purpose of this study was to investigate the relationship between FMIC and diabetes in adulthood using a representative community sample of adults, while considering the role of gender as well as the impact of various potential confounds, detailed below, on these relationships. Our goal was to test two hypotheses: (1) FMIC will be associated with diabetes even after controlling for many of the known diabetes risk factors and (2) the FMIC–diabetes relationship will be stronger for men than for women.

### Diabetes risk factors

Younger adults are more likely to experience FMIC as a result of growing rates of incarceration in the last 40 years.^33^ Also, African Americans and Hispanics experience more FMIC exposure than non-Hispanic Whites.^34^ Gender, race, and age are all related to diabetes risk. Men have a higher prevalence of type 2 diabetes than women, and the prevalence of diabetes tends to peak among individuals aged 65 and older.^3^ Those from historically underrepresented and underserved racial and ethnic groups experience diabetes at greater prevalence compared to non-Hispanic Whites, with the highest risk reported among Aboriginal people.^3^ FMIC is associated with lower levels of education and lower average household income in adulthood.^35,36^ There is likewise an association between lower socioeconomic status (SES) and type 2 diabetes prevalence.^37^ The same relationship exists for educational attainment and risk of type 2 diabetes.^38^

FMIC has been linked to high levels of cigarette smoking in adulthood,^39^ and smoking increases the risk of developing diabetes.^40^ Some research supports a link between FMIC exposure and obesity as well as lower physical activity,^41^ although other research has not observed such associations.^33^ Obesity is known to contribute to type 2 diabetes development.^42^ Sedentary lifestyle is also associated with diabetes, independent of obesity.^43^ FMIC predicts early adulthood depression.^14^ Research has shown a bidirectional association between diabetes and depression.^44^

Children exposed to FMIC experience other difficult circumstances at home, some of which are directly or indirectly related to the incarceration of a family member. Problems with impulse control, substance use problems, and domestic violence are more common among men who ultimately are incarcerated,^45^ and incarceration itself can also lead to the development of substance use disorders and depression.^46^ Parental depression appears to be inversely associated with diabetes-related outcomes in adulthood. For instance, living with a depressed mother or father is associated with having a lower body mass index (BMI) in middle adulthood.^47^ Paternal depression is likewise associated with better glucose control, as indicated by lower glycosylated hemoglobin levels.^47^ To our knowledge, no prior studies have looked specifically at the relationship between parental substance abuse and diabetes risk in adulthood. However, exposure to parental alcoholism has been linked to adult obesity.^48^ Additionally, witnessing parental domestic violence increases the likelihood of being diagnosed with diabetes and obesity in adulthood.^47,49^

Marriage can provide socioemotional support with physical health benefits, particularly for men.^50^ However, to our knowledge, the relationship between marital status and FMIC has not previously been the focus of empirical investigation, thus it is unclear what role FMIC may play in marriage. A large prospective study reported increased risk of type 2 diabetes onset among widowed men compared with married men.^51^ Neither divorced/separated men nor never married men had an elevated risk of incident type 2 diabetes compared with married men.^51^ Widowhood has similarly been shown to be predictive of diabetes status in cross-sectional Australian cohorts.^52^ In contrast, other prospective research has not found marital status to predict diabetes among obese men and women.^53^

Those who are exposed to FMIC likely experience reduced access to a primary doctor or health insurance because FMIC has deleterious socioeconomic effects on the family.^34^ The relationship between medical care access and diabetes may be multifaceted. Previous research has suggested that adults with diabetes have higher rates of health insurance coverage compared to those without diabetes.^54^ However, nationally representative research suggests that an estimated 16% of US adults with known diabetes are uninsured.^55^ Lack of insurance coverage is associated with under-diagnosis of diabetes and poorer diabetes management.^55^

## Methods

### Data source and sample

As has been described elsewhere,^8^ we used data derived from a large representative data set of the CDC’s Behavioral Risk Factor Surveillance System (BRFSS) to test our hypotheses.^56^ Computer-assisted telephone interviews were used to collect data over the phone from a large, representative sample of non-institutionalized adults living in households using telephone landlines and cellular phones across all 50 states, the District of Columbia, and three US territories.^56^

Secondary data analysis of the 2012 optional ACE module was used for this study. The module, adapted from the original CDC-Kaiser ACE study,^7^ was answered by respondents aged 18 and older in five states (response rates): Iowa (56.8%), Tennessee (45.4%), North Carolina (40.4%), Oklahoma (47.8%), and Wisconsin (50.4%).^56^ To focus on adulthood risk for diabetes, data for BRFSS participants aged 40 and older were included. Those with missing data on any of the variables in the analysis were excluded (6.9% missing for men; 6.4% missing for women).

### Measures

#### FMIC exposure

Respondents with a positive history of FMIC before the age of 18 were identified through a response of “once” or “more than once” to the following question: “Did you live with anyone who served time or was sentenced to serve time in a prison, jail, or other correctional facility?” Individuals reporting “never” or “don’t know/not sure” to the latter question were categorized as not experiencing FMIC.

#### Outcome

A history of diabetes was determined by a “yes” response to a question of whether “a doctor, nurse, or other health professional had ever told you had diabetes?” We only counted self-reported diabetes diagnosis. Respondents who reported prediabetes, borderline, or gestational diabetes were coded as “no.”

#### Control variables

Several demographic characteristics were assessed, including age (categorized as 40–64, 65–79, and 80+ years), gender, and race (dichotomized as non-Hispanic White, vs non-White or Hispanic). Education and household income were used to characterize adult SES. Education was assessed based on the following categories: did not graduate high school, graduated high school, attended college or technical school, and graduated from college or technical school. Household income, reported in 2012 dollars, was categorized as less than US$15,000, US$15,000–25,000, US$25,000–50,000, US$50,000–75,000, and above US$75,000.

Childhood risk factors were based on participants’ responses to questions regarding experiences before the age of 18, including parental substance abuse (endorsement of “Did you live with anyone who was a problem drinker or alcoholic?” and/or “Did you live with anyone who used illegal street drugs or who abused prescription medications?”), mentally ill family member (“Did you live with anyone who was depressed, mentally ill, or suicidal?”), exposure to domestic violence (“How often did your parents or adults in your home ever slap, hit, kick, punch, or beat each other up?” dichotomized as never vs once or more), physical abuse (“How often did a parent or adult in your home ever hit, beat, kick, or physically hurt you in any way (excluding spanking)?” dichotomized as ever vs never), sexual abuse (“How often did anyone at least 5 years older than you or an adult, ever touch you sexually?” dichotomized as ever vs never), and verbal abuse (“How often did a parent or adult in your home ever swear at you, insult you, or put you down?” dichotomized as one time or less vs two times or more).

The health risk behaviors controlled in this study were BMI, smoking status, and physical activity level. Self-reported weight in kilograms (kg) was divided by self-reported height in squared meters (m^2^) to define BMI, which was then categorized into ranges defining normal weight (BMI <25 kg/m^2^), overweight (BMI = 25–29.99 kg/m^2^), and obese (BMI = 30 kg/m^2^ or higher). Respondents who endorsed smoking at least 100 cigarettes in their entire life were classified as smokers and those who smoked less than 100 were classified as non-smokers.^57^ Physical activity was dichotomized as having, in the past month, exercised outside of work or not.

Marital status was categorized as being either married or common-law versus being single, divorced, separated, or never married. Depression history was dichotomized based on participant response to the question of whether one had ever been told by a doctor, nurse, or other health professional that he or she had a depressive disorder, including depression, minor depression, dysthymia, or major depression. Finally, healthcare access was measured based on responses to two items: whether or not the respondent had current healthcare coverage insurance, including prepaid or government plans, and how many persons they think of as their “personal doctor or health care provider” (zero vs one or more).

### Statistical analyses

The purpose of the analyses was to determine the odds of diabetes for individuals who reported FMIC. Of particular interest was the degree to which potential confounds might attenuate the relationship between FMIC exposure and diabetes. Logistic regression analyses were conducted separately for men and women, with FMIC as the focal exposure and diabetes as the outcome. We applied a weighting variable that was constructed by the CDC to correct for non-response and likelihood of selection in order for the sample to be representative of community dwellers in each of the five states. This weighting variable was then rescaled to a mean of 1 for the subsample, which is the standardized technique of normalizing weights so as to avoid falsely narrowing the confidence intervals (CIs).

Each model included age and race as well as FMIC. The first model included only age and race. The second model also adjusted for childhood risk factors, the third for health behaviors, the fourth for adult SES, the fifth for depression, the sixth for marital status, the seventh for healthcare access, the eighth for state of residency, and the final model fully adjusted for all the aforementioned variables.

We conducted a sensitivity analysis to determine if the odds of diabetes among those with FMIC varied if we included prediabetes and borderline diabetes, and for women, gestational diabetes in the dependent variable. We found that the estimated odds ratios associated with FMIC were quite comparable. Among men, the odds of the more inclusive diabetes variable were slightly more elevated and remained statistically significant. For example, among men, when prediabetes and borderline diabetes were included in the outcome variable, the odds of diabetes among those with FMIC were 1.70 (95% CI = 1.33, 2.17). When those with prediabetes and borderline diabetes were excluded from the analysis, the odds of diabetes in the fully adjusted model (Model 9) among those with FMIC were 1.64 (95% CI = 1.27, 2.11).

## Results

As shown in Table 1, 16.6% of men and 13.8% of women in the sample had diabetes. Among men, those with diabetes were much more likely to have had FMIC exposure than those without diabetes (7.9% vs 4.8%, p <0.001). Among women, FMIC was not significantly associated with diabetes in the bivariate analysis (p = 0.075). For both women and men, the prevalence of FMIC was higher among younger respondents, Hispanic or non-White respondents, compared to non-Hispanic White, those with less than a high school education, those with lower income, and single/divorced/separated respondents. Those who had been exposed to adverse childhood events had a much higher prevalence of FMIC exposure as well and this was evident for all six forms of childhood adversities examined (i.e. parental substance abuse, parental mental illness, parental domestic violence, childhood physical abuse, childhood sexual abuse, childhood verbal abuse). Ever smokers, those with depressive disorders, and those who were without healthcare coverage or a personal doctor also had a higher prevalence of FMIC. Of the five states included in this analysis, the prevalence of FMIC was highest in Tennessee. Women who were obese and who did not exercise regularly also reported a higher prevalence of FMIC exposure, but these two factors were not statistically significant for men.

**Table 1.** Unweighted sample sizes and weighted percentages of males and females in the adverse childhood experiences module of the 2012 BRFSS.

Sample characteristics	Men (n = 8790)		p-value	Women (n = 14,255)		p-value
	Total %	% who had FMIC		Total %	% who had FMIC	
Diabetes			<0.001			0.075
Yes (not borderline or gestational)	16.6	7.9		13.8	5.4	
No	83.4	4.8		86.2	4.5	
*Demographics*						
Age			<0.001			<0.001
40–64 years	73.0	6.3		68.0	5.8	
65–79 years	22.2	2.8		24.4	2.5	
80+ years	4.9	1.6		7.5	1.7	
Race			<0.001			<0.001
Non-Hispanic White	81.4	4.5		82.8	4.0	
Hispanic or non-White	18.6	8.8		17.2	8.0	
Education			<0.001			<0.001
Less than high school	15.0	12.8		12.8	8.9	
High school graduate	30.8	4.2		32.0	4.5	
Some college or technical school	28.9	4.9		31.9	4.9	
College or technical school graduate	25.3	2.6		23.3	2.2	
Household income			<0.001			<0.001
<US$15,000	8.5	12.7		9.6	9.0	
US$15,000–24,999	14.4	8.4		16.5	7.8	
US$25,000–49,999	25.3	5.2		23.5	4.6	
US$50,000–74,999	15.8	4.2		13.9	3.7	
⩾US$75,000	26.3	2.2		20.3	2.4	
Do not know/refused/missing	9.7	4.9		16.2	2.7	
Marital status			<0.001			<0.001
Married/common-law	70.1	4.2		60.7	3.5	
Single/divorced/separated	29.9	7.9		39.3	6.5	
*Adverse childhood events*						
Parent abused drugs or alcohol			<0.001			<0.001
Yes	23.0	15.7		26.6	13.5	
No	77.0	2.2		73.4	1.4	
Lived with mentally ill household member in childhood			<0.001			<0.001
Yes	10.2	14.5		15.0	12.0	
No	89.8	4.2		85.0	3.3	
Parental domestic violence in childhood			<0.001			<0.001
Yes	15.5	14.3		16.2	14.1	
No	84.5	3.6		83.8	2.8	
Physical abuse			<0.001			<0.001
Yes	13.4	14.5		13.5	12.9	
No	86.6	3.9		86.5	3.4	
Verbal abuse			<0.001			<0.001
Yes	22.3	11.4		22.8	10.5	
No	77.7	3.6		77.2	2.9	
Sexual abuse			<0.001			<0.001
Yes	1.9	17.4		5.4	23.5	
No	98.1	5.1		94.6	3.6	
*Health*						
Smoking status			<0.001			<0.001
Smoked ⩾100 cigarettes	57.7	6.7		43.8	7.1	
Never smoked 100 cigarettes	42.3	3.3		56.2	2.8	
Exercised in the past month			0.467			<0.001
Yes	73.1	5.2		71.0	4.0	
No	26.9	5.6		29.0	6.2	
Body mass index category			0.279			<0.001
Not overweight/obese	21.0	4.9		33.2	3.3	
Overweight	43.7	5.0		30.0	4.1	
Obese	34.5	5.9		30.4	7.0	
Do not know/refused/missing	0.8	5.8		6.4	3.3	
Depressive disorder			<0.001			<0.001
Yes	15.0	10.5		22.0	8.0	
No	85.0	4.4		78.0	3.7	
Healthcare coverage			<0.001			<.001
Yes	88.3	4.5		89.4	4.1	
No	11.7	11.2		10.6	8.9	
Has personal doctor			<0.001			<0.001
Yes	84.3	4.7		91.3	4.4	
No	15.7	8.6		8.7	7.8	
State of residency			<0.001			0.001
Iowa	11.2	4.0		11.6	3.7	
North Carolina	37.2	4.7		37.0	4.9	
Oklahoma	7.4	5.1		7.2	4.5	
Tennessee	22.4	7.8		22.5	5.7	
Wisconsin	21.8	4.3		21.6	3.6	

BRFSS: Behavioral Risk Factor Surveillance System; FMIC: family member incarceration during childhood.

**Figure 1.**
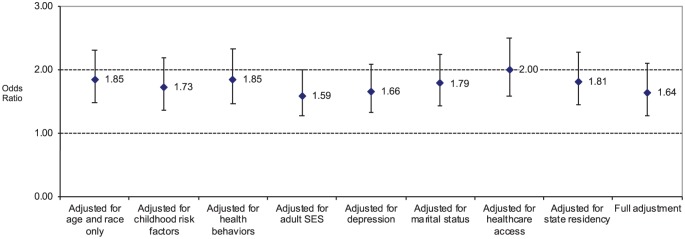
Odds ratio and 95% confidence interval of diabetes among males reporting family member incarceration during childhood. All data are adjusted for age and race. Sample size n = 8790 in all models.

As shown in Figure 1, across nine different models, FMIC exposure was robustly associated with elevated odds of diabetes among men. These odds ranged from a low of 1.59 (for the model adjusting for adult SES) to a high of 2.00 (for the model adjusting for healthcare access). In the fully adjusted model that included all of the variables in the previous eight models, the odds of diabetes were 1.64 for those reporting FMIC (p <0.001).

**Figure 2.**
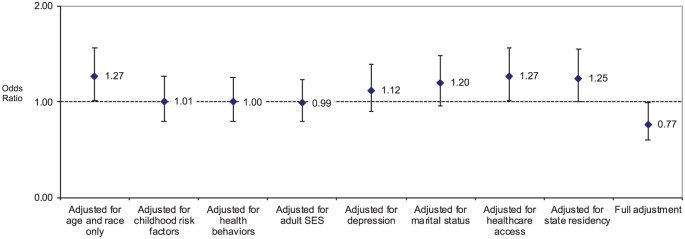
Odds ratio and 95% confidence interval of diabetes among females reporting family member incarceration during childhood. All data are adjusted for age and race. Sample size n = 14,255 in all models.

As shown in Figure 2, for women, the first eight logistic regression models hovered around 1, ranging from 0.99 to 1.27. In the fully adjusted model, which took into account 18 variables, the odds declined to 0.77.

Table 2 provides two logistic regression models for each gender: the first logistic regression adjusts for age and race only (Model 1 in Figures 1 and 2), and the second provides the fully adjusted model (Model 9 in Figures 1 and 2). Table 2 shows a large number of characteristics associated with diabetes in both men and women. These include older age, Hispanic or non-White ethnicity, lower income, lower levels of exercise, obesity, lifetime history of depression, and not having a personal healthcare professional. Once all the lifestyle and socioeconomic characteristics were taken into account concurrently in the fully adjusted logistic regression analyses, neither marital status, smoking history, nor the six childhood adversity variables (i.e. parental substance abuse, parental mental illness, parental domestic violence, childhood physical abuse, childhood sexual abuse, childhood verbal abuse) were statistically significant for men or women.

**Table 2.** Gender-specific logistic regression models of diabetes among respondents reporting versus never reporting family member incarceration during childhood of the BRFSS 2012.

Model	Men (n = 8790)				Women (n = 14,255)			
	Age-race adjusted	p-value	Fully adjusted	p-value	Age-race adjusted	p-value	Fully adjusted	p-value
	OR (95% CI)		OR (95% CI)		OR (95% CI)		OR (95% CI)	
*Key variable of interest*								
FMIC	1.85 (1.48–2.31)	<0.001	1.64 (1.27–2.11)	<0.001	1.27 (1.02–1.57)	0.032	0.77 (0.61–0.99)	0.039
*Control variables*								
Age								
40–64	1.00 (Ref.)		1.00 (Ref.)		1.00 (Ref.)		1.00 (Ref.)	
65–79	2.27 (2.00–2.57)	0.001	2.21 (1.91–2.55)	0.001	2.06 (1.85–2.29)	0.001	1.87 (1.66–2.12)	0.001
⩾80	1.88 (1.47–2.40)	<0.001	2.01 (1.53–2.63)	0.001	1.83 (1.54–2.18)	0.001	2.01 (1.65–2.46)	0.001
Race								
Non-Hispanic White	1.00 (Ref.)		1.00 (Ref.)		1.00 (Ref.)		1.00 (Ref.)	
Hispanic or non-White	1.63 (1.42–1.87)	<0.001	1.47 (1.26–1.71)	<0.001	1.89 (1.68–2.12)	<0.001	1.44 (1.26–1.64)	<0.001
Education								
Below high school			1.13 (0.90–1.41)	0.298			1.56 (1.28–1.89)	<0.001
High school graduate			1.04 (0.87–1.25)	0.654			1.23 (1.04–1.45)	0.015
Some college or technical school			1.06 (0.89–1.27)	0.530			1.19 (1.01–1.41)	0.034
College or technical school graduate			1.00 (Ref.)				1.00 (Ref.)	
Household income								
No or <US$15,000			2.14 (1.62–2.81)	<0.001			3.14 (2.46–4.01)	0.001
US$15,000–25,000			2.39 (1.91–2.99)	<0.001			2.53 (2.03–3.15)	0.001
US$25,000–50,000			1.45 (1.19–1.76)	<0.001			2.03 (1.66–2.49)	0.001
US$50,000–75,000			1.41 (1.14–1.74)	0.001			1.23 (0.97–1.55)	0.083
⩾US$75,000			1.00 (Ref.)				1.00 (Ref.)	
Missing data			1.23 (0.95–1.58)	0.118			1.95 (1.57–2.43)	0.001
*Childhood adversity*								
Physical abuse								
No			1.00 (Ref.)				1.00 (Ref.)	
Yes			0.90 (0.74–1.11)	0.329			1.13 (0.95–1.35)	0.157
Sexual abuse								
No			1.00 (Ref.)				1.00 (Ref.)	
Yes			0.85 (0.54–1.33)	0.476			1.19 (0.96–1.47)	0.117
Verbal abuse								
No			1.00 (Ref.)				1.00 (Ref.)	
Yes			1.14 (0.96–1.35)	0.136			1.09 (0.94–1.27)	0.243
Parental substance abuse								
No			1.00 (Ref.)				1.00 (Ref.)	
Yes			0.96 (0.81–1.13)	0.607			0.90 (0.79–1.03)	0.118
Lived with mentally ill								
No			1.00 (Ref.)				1.00 (Ref.)	
Yes			0.84 (0.68–1.05)	0.126			1.18 (1.01–1.38)	0.038
Domestic violence								
No			1.00 (Ref.)				1.00 (Ref.)	
Yes			1.13 (0.93–1.36)	0.219			0.97 (0.82–1.13)	0.655
*Adult health*								
Smoker								
Never smoked 100			1.00 (Ref.)				1.00 (Ref.)	
Smoked 100 or more			0.91 (0.80–1.04)	0.170			0.98 (0.88–1.09)	0.737
Exercised in past month								
Yes			1.00 (Ref.)				1.00 (Ref.)	
No			1.17 (1.02–1.33)	0.025			1.36 (1.22–1.51)	0.001
Body mass index category								
Not overweight or obese			1.00 (Ref.)				1.00 (Ref.)	
Overweight			1.63 (1.34–1.98)	0.001			2.11 (1.80–2.47)	0.001
Obese			4.53 (3.75–5.48)	0.001			5.17 (4.45–6.01)	0.001
Missing/refused/etc.			1.80 (0.87–3.76)	0.115			3.10 (2.47–3.90)	0.001
Marital status								
Single/divorced/separated/never married			1.00 (Ref.)				1.00 (Ref.)	
Married			0.90 (0.78–1.03)	0.135			1.00 (0.89–1.13)	0.959
Depressive disorder								
No			1.00 (Ref.)				1.00 (Ref.)	
Yes			1.79 (1.52–2.11)	0.001			1.46 (1.29–1.65)	0.001
*Healthcare*								
Healthcare coverage								
Yes			1.28 (1.01–1.62)	0.044			1.18 (0.99–1.42)	0.070
No			1.00 (Ref.)				1.00 (Ref.)	
Number of healthcare professionals								
0			1.00 (Ref.)				1.00 (Ref.)	
⩾1			3.06 (2.38–3.93)	0.001			2.35 (1.86–2.98)	0.001
State of residence								
Iowa			1.00 (Ref.)				1.00 (Ref.)	
North Carolina			1.09 (0.88–1.34)	0.446			1.13 (0.94–1.35)	0.196
Oklahoma			1.41 (1.07–1.85)	0.014			1.05 (0.83–1.35)	0.673
Tennessee			1.15 (0.92–1.43)	0.232			1.27 (1.06–1.54)	0.011
Wisconsin			0.76 (0.60–0.96)	0.021			0.94 (0.77–1.14)	0.514
−2 Log-likelihood	7695.1		6925.6		11,176.2		9952.4	
Nagelkerke *R*^2^	0.041		0.178		0.034		0.180	

OR: odds ratio; CI: confidence interval; BRFSS: Behavioral Risk Factor Surveillance System; FMIC: family member incarceration during childhood.

In comparison to respondents from Iowa, women from Oklahoma had higher odds of diabetes, and women from Wisconsin had lower odds. Among men, only respondents from Tennessee had significantly higher odds of diabetes than men from Iowa. The more detailed information on the association between FMIC and diabetes for each of the nine logistic regression analyses is provided in Figures 1 and 2.

## Discussion

The current investigation sought to examine the impact of an increasingly common yet insufficiently examined early adverse experience, family member incarceration during one’s childhood (FMIC), on the development of diabetes mellitus, a prevalent, chronic disease in later life that is among the leading causes of mortality and is associated with inflammation and metabolic dysfunction. Based on a large data set with representative data from five states, and consistent with predictions, we found that the age-race adjusted odds of diabetes were higher for men exposed to FMIC compared to those who had not experienced that childhood adversity.

After adjustment for 18 risk factors that included age, ethnicity, childhood risk factors (i.e. physical abuse, sexual abuse, verbal abuse, parental substance abuse, parental mental illness, and parental domestic violence), as well as adult SES (i.e. income and education), health behaviors (i.e. physical activity, smoking, body mass), marital status, depression, and access to healthcare (i.e. health insurance, personal healthcare provider), the odds of diabetes among those exposed to FMIC in comparison to those not exposed to FMIC remained significantly high. This finding is consistent with the view of life-course and biological-embedding models that suggest exposure to early adversities at critical developmental stages disrupts the development of systems involved in stress and inflammation responses,^15,18^ including insulin resistance and metabolic dysfunction implicated in the etiology of diabetes.^19,20^

In contrast to men, the odds of diabetes for women hovered around 1.0 for all of the different analyses. An odds ratio of 1.0 indicates that those with FMIC have comparable odds of diabetes to those without. This observed gender difference fits prior evidence suggesting men may be more vulnerable biologically to early adversities than women.^25,28^ They may experience stress-related testosterone suppression, which is linked to insulin resistance.^28^ Furthermore, incarceration frequently interferes with fathers’ contact with children, which may particularly impact their sons’ coping with stress,^29,31^ and boys and men are less likely than girls and women to seek psychosocial support in response to adverse events.^58^

The findings of this study should be interpreted in light of several limitations. First, data are cross-sectional, thus we are unable to draw causal inferences regarding the relationship between FMIC and diabetes. We are not able to determine what aspects of FMIC-exposed households contribute to boys’ negative long-term health. Both our exposure (FMIC) and outcome (diabetes) variables were measured via self-report of exposure and diagnosis, respectively. Notably, prior research supports the accuracy of self-report of diabetes, including high agreement with medical records.^59^ Although underreporting of FMIC should bias our finding toward the null, external validation of reporting via review of records, including the nature of the crime leading to incarceration, as well as its timing, duration, and frequency would be desirable.^34^

Due to limitations in the BRFSS database, we were unable to control for certain known diabetes risk factors, including diabetic family history, nutritional influences, or serum cholesterol level. With regard to sociodemographic factors, we unfortunately could not control for childhood SES or parental education, which are related to parental incarceration and higher diabetes risk.^37,60^ Notable disparities exist among racial and ethnic groups in US incarceration rates,^33^ and while we controlled for race, due to power limitations we unfortunately were not able to perform race-specific analyses. A further limitation of the BRFSS data is the absence of information regarding the relationship of the incarcerated family member to the respondent, the age at which the FMIC exposure occurred, as well as the duration of the incarceration. Finally, we did not have information on the gender-specific response rate for each state. It is possible that gendered variation in response rate and/or different response rates by state may have biased the results. Because the response rates may have varied by gender, state, ethnicity, diabetes, and so on, all findings must be interpreted with caution.

As an investigation into how a particular early adversity may impact later development of a serious health condition, this study also has some noteworthy strengths. It is, to our knowledge, the first study to examine the potential impact of FMIC exposure on diabetes with gender-specific analysis using representative data from disparate states. Our findings are consistent with recent evidence for an association between FMIC and myocardial infarction for sons but not for daughters and support the view that, as an ACE, FMIC is linked to important lasting physical health impacts in men.^8^ In contrast, among women, FMIC was not a major factor associated with diabetes in adulthood, potentially due to gender differences in psychosocial and biological responses to FMIC, in particular, disruption of contact from fathers, although these proposed mechanisms require further direct investigation.

## Conclusion and implications

In this large representative community sample, we found an association between higher odds of diabetes among FMIC-exposed men compared to those without exposure to this form of early adversity. FMIC was not associated with diabetes in women. This research provides a helpful profile of those most susceptible which may be informative for future targeted outreach and intervention.

With regard to criminal justice policy, our findings suggest that the dramatic increase in recent decades of incarceration, particularly in the United States,^11^ may have detrimental long-term health effects for individuals exposed to FMIC persisting into later life. These impacts extend beyond previously identified effects on family stability and psychopathology^13^ and lend support to consideration of alternatives to current incarceration policies and practices, such as investment in diversion strategies to redirect individuals to community-based rehabilitative programs,^61^ facilitating family contact by placing incarcerated individuals in facilities close to their communities,^62,63^ and eliminating visitation policies that create excessive burden for family members, such as restrictive visitation hours and prohibitive fees for visitor background checks or for phone calls.^64^ Future research should examine the impacts of changing incarceration patterns, such as the growth of the US women’s prison population, on children’s long-term health.

These findings also have important implications for policies and practices aimed at reducing health inequities in vulnerable populations. With regard to clinical practice, our results suggest that early identification, assessment, and intervention with youth exposed to FMIC, particularly boys, may be beneficial. Given that FMIC appears to confer higher odds of diabetes and other chronic health conditions, screening for FMIC among adults presenting with diabetes may facilitate early detection and/or prevention of other adverse health outcomes. Furthermore, the development, implementation, and dissemination of empirically supported interventions designed to mitigate the health impacts of FMIC exposure among older adults should be investigated in future research.

## Supplemental Material

2012_BRFSS – Supplemental material for Is exposure to family member incarceration during childhood linked to diabetes in adulthood? Findings from a representative community sample Click here for additional data file. Supplemental material, 2012_BRFSS for Is exposure to family member incarceration during childhood linked to diabetes in adulthood? Findings from a representative community sample by Bradley A White, Keri J West and Esme Fuller-Thomson in SAGE Open Medicine
