# Supplementary material for: Is exposure to family member incarceration during childhood linked to diabetes in adulthood? Findings from a representative community sample
Source: SAGE Open Med. 2020 Feb 5;8:2050312120905165. doi: 10.1177/2050312120905165 (PMC7003170; doi:10.1177/2050312120905165)
Supplement: 2012_BRFSS – Supplemental material for Is exposure to family member incarceration during childhood linked to diabetes in adulthood? Findings from a representative community sample [file 2012_BRFSS.pdf]

BRFSS

2012

Behavioral Risk Factor Surveillance System Questionnaire

January 6, 2012

Behavioral Risk Factor Surveillance System
2012 Questionnaire

Table of Contents

Table of Contents 2
Interviewer's Script 4
Core Sections 7
Section 1: Health Status 7
Section 2: Healthy Days — Health-Related Quality of Life 7
Section 3: Health Care Access 8
Section 4: Exercise 9
Section 5: Chronic Health Conditions 9
Section 6: Oral Health 12
Section 7: Demographics 13
Section 8: Disability 18
Section 9: Tobacco Use 19
Section 10: Alcohol Consumption 20
Section 11: Immunization 21
Section 12: Falls 22
Section 13: Seatbelt Use 22
Section 14: Drinking and Driving 23
Section 15: Breast and Cervical Cancer Screening 23
Section 16: Prostate Cancer Screening 25
Section 17: Colorectal Cancer Screening 26
Section 18: HIV/AIDS 28
Optional Modules 30
Module 1: Pre-Diabetes 30
Module 2: Diabetes 30
Module 3: Healthy Days (Symptoms) 33
Module 4: Visual Impairment and Access to Eye Care 34
Module 5: Sugar Sweetened Beverages and Menu Labeling 37
Module 6: Excess Sun Exposure 38
Module 7: Inadequate Sleep 38
Module 8: Fruits and Vegetables 39
Module 9: Adult Asthma History 43
Module 10: High Risk/Health Care Worker 46
Module 11: Shingles (Zostavax or ZOS) 47
Module 12: Tetanus Diphtheria (Adults) 47
Module 13: Adult Human Papilloma Virus (HPV) 48
Module 14: Prostate Cancer Screening Decision Making Module 49
Module 15: Cancer Survivorship 49
Module 16: Reactions to Race 54
Module 17: Mental Illness and Stigma 56
Module 18: Social Context 58
Module 19: General Preparedness 60
Module 20: Veteran's Health 63
Module 21: Chronic Obstructive Pulmonary Disease (COPD) 65
Module 22: Adverse Childhood Experience 66
Module 23: Random Child Selection 68

Module 24: Childhood Asthma Prevalence 70
Module 25: Childhood Immunization 71
Module 26: HIV/AIDS........ 71
Module 27: Emotional Support and Life Satisfaction........ 72
List of Health Problems to Accompany Module 10, Question 3 74

Interviewer's Script

HELLO, I am calling for the **__(health department)_.** My name is **_____(name)____.** We are gathering information about the health of **__(state)__** residents. This project is conducted by the health department with assistance from the Centers for Disease Control and Prevention. Your telephone number has been chosen randomly, and I would like to ask some questions about health and health practices.

Is this **__(phone number)__** ?

If "No"

Thank you very much, but I seem to have dialed the wrong number. It's possible that your number may be called at a later time. **STOP**

Is this a private residence in **__(state)__** ?

If "Yes" **[Go to cellular phone question]**

If "No" **[Go to college housing]**

Interviewer Note: Telephone service over the internet counts as landline service (includes Vonage, Magic Jack and other home-based phone services.).

College Housing

Do you live in college housing?

Read only if necessary: "By college housing we mean dormitory, graduate student or visiting faculty housing, or other housing arrangements provided by a college or university"

If "No,"

Thank you very much, but we are only interviewing persons who live in a private residence or college housing at this time. **STOP**

Cellular Phone

Is this a cellular telephone?

[Read only if necessary: "By cellular (or cell) telephone we mean a telephone that is mobile and usable outside of your neighborhood."

If "Yes"

Thank you very much, but we are only interviewing land line telephones and private residences or college housing. **STOP**

CATI NOTE: IF (College Housing = Yes) continue; otherwise go to Adult Random Selection

Adult

Are you 18 years of age or older?

1 **Yes, respondent is male** **[Go to Page 6]**
2 **Yes, respondent is female** **[Go to Page 6]**
3 **No**

If "No",

Thank you very much, but we are only interviewing persons aged 18 or older at this time. **STOP**

Adult Random Selection

I need to randomly select one adult who lives in your household to be interviewed. How many members of your household, including yourself, are 18 years of age or older?

__ Number of adults

If "1,"
Are you the adult?

If "yes,"
Then you are the person I need to speak with. Enter 1 man or 1 woman below (Ask gender if necessary). **Go to page 6.**

If "no,"
Is the adult a man or a woman? Enter 1 man or 1 woman below. May I speak with **[fill in (him/her) from previous question]**? **Go to "correct respondent" on the next page.**

How many of these adults are men and how many are women?

__ Number of men

__ Number of women

The person in your household that I need to speak with is _______________.

If "you," go to page 6

To the correct respondent:

HELLO, I am calling for the **__(health department)__.** My name is **_____(name)____.** We are gathering information about the health of **__(state)__** residents. This project is conducted by the health department with assistance from the Centers for Disease Control and Prevention. Your telephone number has been chosen randomly, and I would like to ask some questions about health and health practices.

Core Sections

I will not ask for your last name, address, or other personal information that can identify you. You do not have to answer any question you do not want to, and you can end the interview at any time. Any information you give me will be confidential. If you have any questions about the survey, please call **(give appropriate state telephone number)**.

Section 1: Health Status

1.1 Would you say that in general your health is—

(73)

Please read:

1 Excellent
2 Very good
3 Good
4 Fair

Or

5 Poor

Do not read:

7 Don't know / Not sure
9 Refused

Section 2: Healthy Days — Health-Related Quality of Life

2.1 Now thinking about your physical health, which includes physical illness and injury, for how many days during the past 30 days was your physical health not good?

(74–75)

_ _ Number of days
8 8 None
7 7 Don't know / Not sure
9 9 Refused

2.2 Now thinking about your mental health, which includes stress, depression, and problems with emotions, for how many days during the past 30 days was your mental health not good?

(76–77)

_ _ Number of days
8 8 None **[If Q2.1 and Q2.2 = 88 (None), go to next section]**
7 7 Don't know / Not sure
9 9 Refused

2.3 During the past 30 days, for about how many days did poor physical or mental health keep you from doing your usual activities, such as self-care, work, or recreation?

(78-79)

_ _ Number of days
8 8 None
7 7 Don't know / Not sure
9 9 Refused

Section 3: Health Care Access

3.1 Do you have any kind of health care coverage, including health insurance, prepaid plans such as HMOs, government plans such as Medicare, or Indian Health Service?

(80)

1 Yes
2 No
7 Don't know / Not sure
9 Refused

3.2 Do you have one person you think of as your personal doctor or health care provider?

If "No," ask: "Is there more than one, or is there no person who you think of as your personal doctor or health care provider?"

(81)

1 Yes, only one
2 More than one
3 No
7 Don't know / Not sure
9 Refused

3.3 Was there a time in the past 12 months when you needed to see a doctor but could not because of cost?

(82)

1 Yes
2 No
7 Don't know / Not sure
9 Refused

3.4 About how long has it been since you last visited a doctor for a routine checkup? A routine checkup is a general physical exam, not an exam for a specific injury, illness, or condition.

(83)

1 Within the past year (anytime less than 12 months ago)
2 Within the past 2 years (1 year but less than 2 years ago)
3 Within the past 5 years (2 years but less than 5 years ago)
4 5 or more years ago
7 Don't know / Not sure
8 Never
9 Refused

Section 4: Exercise

4.1 During the past month, other than your regular job, did you participate in any physical activities or exercises such as running, calisthenics, golf, gardening, or walking for exercise?

(84)

1 Yes
2 No
7 Don't know / Not sure
9 Refused

Section 5: Chronic Health Conditions

Now I would like to ask you some questions about general health conditions.

Has a doctor, nurse, or other health professional EVER told you that you had any of the following? For each, tell me "Yes," "No," or you're "Not sure."

5.1 (Ever told) you that you had a heart attack also called a myocardial infarction?

(85)

1 Yes
2 No
7 Don't know / Not sure
9 Refused

5.2 (Ever told) you had angina or coronary heart disease?

(86)

1 Yes
2 No
7 Don't know / Not sure
9 Refused

5.3 (Ever told) you had a stroke?

(87)

1 Yes
2 No
7 Don't know / Not sure
9 Refused

5.4 (Ever told) you had asthma?

(88)

1 Yes
2 No **[Go to Q5.6]**
7 Don't know / Not sure **[Go to Q5.6]**
9 Refused **[Go to Q5.6]**

5.5 Do you still have asthma?

(89)

1 Yes
2 No
7 Don't know / Not sure
9 Refused

5.6 (Ever told) you had skin cancer?

(90)

1 Yes
2 No
7 Don't know / Not sure
9 Refused

5.7 (Ever told) you had any other types of cancer?

(91)

1 Yes
2 No
7 Don't know / Not sure
9 Refused

5.8 (Ever told) you have Chronic Obstructive Pulmonary Disease or COPD, emphysema or chronic bronchitis?

(92)

1 Yes
2 No
7 Don't know / Not sure
9 Refused

5.9 (Ever told) you have some form of arthritis, rheumatoid arthritis, gout, lupus, or fibromyalgia?

(93)

1 Yes
2 No
7 Don't know / Not sure
9 Refused

INTERVIEWER NOTE: Arthritis diagnoses include:

- rheumatism, polymyalgia rheumatica
- osteoarthritis (not osteoporosis)
- tendonitis, bursitis, bunion, tennis elbow
- carpal tunnel syndrome, tarsal tunnel syndrome
- joint infection, Reiter's syndrome
- ankylosing spondylitis; spondylosis
- rotator cuff syndrome
- connective tissue disease, scleroderma, polymyositis, Raynaud's syndrome
- vasculitis (giant cell arteritis, Henoch-Schonlein purpura, Wegener's granulomatosis,
- polyarteritis nodosa)

5.10 (Ever told) you have a depressive disorder, including depression, major depression, dysthymia, or minor depression?

(94)

1 Yes
2 No
7 Don't know / Not sure
9 Refused

5.11 (Ever told) you have kidney disease? Do NOT include kidney stones, bladder infection or incontinence.

INTERVIEWER NOTE: Incontinence is not being able to control urine flow.

(95)

1 Yes
2 No
7 Don't know / Not sure
9 Refused

5.12 Do you have any trouble seeing, even when wearing glasses or contact lenses?

(96)

1 Yes
2 No
3 Not applicable (blind)
7 Don't know / Not sure
9 Refused

5.13 (Ever told) you have diabetes? (97)

If "Yes" and respondent is female, ask: "Was this only when you were pregnant?"

If respondent says pre-diabetes or borderline diabetes, use response code 4.

1 Yes
2 Yes, but female told only during pregnancy
3 No
4 No, pre-diabetes or borderline diabetes
7 Don't know / Not sure
9 Refused

CATI note: If Q5.13 = 1 (Yes), go to Diabetes Optional Module (if used). If any other response to Q5.13, go to Pre-Diabetes Optional Module (if used), otherwise, go to next section.

Section 6: Oral Health

6.1 How long has it been since you last visited a dentist or a dental clinic for any reason? Include visits to dental specialists, such as orthodontists. (98)

Read only if necessary:

1 Within the past year (anytime less than 12 months ago)
2 Within the past 2 years (1 year but less than 2 years ago)
3 Within the past 5 years (2 years but less than 5 years ago)
4 5 or more years ago

Do not read:

7 Don't know / Not sure
8 Never
9 Refused

6.2 How many of your permanent teeth have been removed because of tooth decay or gum disease? Include teeth lost to infection, but do not include teeth lost for other reasons, such as injury or orthodontics.

NOTE: If wisdom teeth are removed because of tooth decay or gum disease, they should be included in the count for lost teeth.

(99)

1 1 to 5
2 6 or more but not all
3 All
8 None
7 Don't know / Not sure
9 Refused

Section 7: Demographics

7.1 What is your age? (100-101)

_ _ Code age in years
0 7 Don't know / Not sure
0 9 Refused

7.2 Are you Hispanic or Latino? (102)

1 Yes
2 No
7 Don't know / Not sure
9 Refused

7.3 Which one or more of the following would you say is your race? (103 -108)

(Check all that apply)

Please read:

1 White
2 Black or African American
3 Asian
4 Native Hawaiian or Other Pacific Islander
5 American Indian or Alaska Native

Or

6 Other [specify]_______________

Do not read:

8 No additional choices
7 Don't know / Not sure
9 Refused

CATI note: If more than one response to Q7.3; continue. Otherwise, go to Q7.5.

7.4 Which one of these groups would you say best represents your race? (109)

Please read:

1 White
2 Black or African American
3 Asian
4 Native Hawaiian or Other Pacific Islander
5 American Indian or Alaska Native

Or

6 Other [specify]_______________

Do not read:

7 Don't know / Not sure
9 Refused

7.5 Have you ever served on active duty in the United States Armed Forces, either in the regular military or in a National Guard or military reserve unit? Active duty does not include training for the Reserves or National Guard, but DOES include activation, for example, for the Persian Gulf War.

(110)

1 Yes
2 No

Do not read:

7 Don't know / Not sure
9 Refused

7.6 Are you…?

(111)

Please read:

1 Married
2 Divorced
3 Widowed
4 Separated
5 Never married

Or

6 A member of an unmarried couple

Do not read:

9 Refused

7.7 How many children less than 18 years of age live in your household?

(112-113)

_ _ Number of children
8 8 None
9 9 Refused

7.8 What is the highest grade or year of school you completed?

(114)

Read only if necessary:

1 Never attended school or only attended kindergarten
2 Grades 1 through 8 (Elementary)
3 Grades 9 through 11 (Some high school)
4 Grade 12 or GED (High school graduate)
5 College 1 year to 3 years (Some college or technical school)
6 College 4 years or more (College graduate)

Do not read:

9 Refused

7.9 Are you currently…?

(115)

Please read:

1 Employed for wages
2 Self-employed
3 Out of work for more than 1 year
4 Out of work for less than 1 year
5 A Homemaker
6 A Student
7 Retired

Or

8 Unable to work

Do not read:

9 Refused

7.10 Is your annual household income from all sources—

(116-117)

If respondent refuses at ANY income level, code '99' (Refused)

Read only if necessary:

0 4 Less than $25,000 **If "no," ask 05; if "yes," ask 03**
($20,000 to less than $25,000)

0 3 Less than $20,000 **If "no," code 04; if "yes," ask 02**
($15,000 to less than $20,000)

0 2 Less than $15,000 **If "no," code 03; if "yes," ask 01**
($10,000 to less than $15,000)

0 1 Less than $10,000 **If "no," code 02**

0 5 Less than $35,000 **If "no," ask 06**
($25,000 to less than $35,000)

0 6 Less than $50,000 **If "no," ask 07**
($35,000 to less than $50,000)

0 7 Less than $75,000 **If "no," code 08**
($50,000 to less than $75,000)

0 8 $75,000 or more

Do not read:

7 7 Don't know / Not sure
9 9 Refused

7.11 About how much do you weigh without shoes? (118-121)

NOTE: If respondent answers in metrics, put "9" in column 118.

Round fractions up

_ _ _ _ Weight *(pounds/kilograms)*
7 7 7 7 Don't know / Not sure
9 9 9 9 Refused

7.12 About how tall are you without shoes? (122-125)

NOTE: If respondent answers in metrics, put "9" in column 122.

Round fractions down

_ _ **/** _ _ Height *(f t / inches/meters/centimeters)*
7 7/ 7 7 Don't know / Not sure
9 9/ 9 9 Refused

7.13 What county do you live in? (126-128)

_ _ _ ANSI County Code (formerly FIPS county code)
7 7 7 Don't know / Not sure
9 9 9 Refused

7.14 What is the ZIP Code where you live? (129-133)

_ _ _ _ _ ZIP Code
7 7 7 7 7 Don't know / Not sure
9 9 9 9 9 Refused

7.15 Do you have more than one telephone number in your household? Do not include cell phones or numbers that are only used by a computer or fax machine. (134)

1	Yes	
2	No	**[Go to Q7.17]**
7	Don't know / Not sure	**[Go to Q7.17]**
9	Refused	**[Go to Q7.17]**

7.16 How many of these telephone numbers are residential numbers?

(135)

_	Residential telephone numbers **[6 = 6 or more]**	
7	Don't know / Not sure	
9	Refused	

7.17 Do you have a cell phone for personal use? Please include cell phones used for both business and personal use.

(136)

1	Yes	
2	No	**[Go to Q7.19]**
7	Don't know / Not sure	**[Go to Q7.19]**
9	Refused	**[Go to Q7.19]**

7.18 Thinking about all the phone calls that you receive on your landline and cell phone, what percent, between 0 and 100, are received on your cell phone?

(137-139)

_ _ _	Enter percent (1 to 100)
8 8 8	Zero
7 7 7	Don't know / Not sure
9 9 9	Refused

7.19 Do you own or rent your home?

(140)

1	Own
2	Rent
3	Other arrangement
7	Don't know / Not sure
9	Refused

INTERVIEWER NOTE: "Other arrangement" may include group home, staying with friends or family without paying rent.

NOTE: Home is defined as the place where you live most of the time/the majority of the year.

7.20 Indicate sex of respondent. **Ask only if necessary.**

(141)

1 Male **[Go to next section]**
2 Female **[If respondent is 45 years old or older, go to next section]**

7.21 To your knowledge, are you now pregnant?

(142)

1 Yes
2 No
7 Don't know / Not sure
9 Refused

Section 8: Disability

The following questions are about health problems or impairments you may have.

8.1 Are you limited in any way in any activities because of physical, mental, or emotional problems?

(143)

1 Yes
2 No
7 Don't know / Not Sure
9 Refused

8.2 Do you now have any health problem that requires you to use special equipment, such as a cane, a wheelchair, a special bed, or a special telephone?

(144)

Include occasional use or use in certain circumstances.

1 Yes
2 No
7 Don't know / Not Sure
9 Refused

Section 9: Tobacco Use

9.1 Have you smoked at least 100 cigarettes in your entire life?

(145)

NOTE: 5 packs = 100 cigarettes

1	Yes	
2	No	**[Go to Q9.5]**
7	Don’t know / Not sure	**[Go to Q9.5]**
9	Refused	**[Go to Q9.5]**

9.2 Do you now smoke cigarettes every day, some days, or not at all?

(146)

1	Every day	
2	Some days	
3	Not at all	**[Go to Q9.4]**
7	Don’t know / Not sure	**[Go to Q9.5]**
9	Refused	**[Go to Q9.5]**

9.3 During the past 12 months, have you stopped smoking for one day or longer because you were trying to quit smoking?

(147)

1	Yes	**[Go to Q9.5]**
2	No	**[Go to Q9.5]**
7	Don’t know / Not sure	**[Go to Q9.5]**
9	Refused	**[Go to Q9.5]**

9.4 How long has it been since you last smoked a cigarette, even one or two puffs?

(148-149)

0 1	Within the past month (less than 1 month ago)
0 2	Within the past 3 months (1 month but less than 3 months ago)
0 3	Within the past 6 months (3 months but less than 6 months ago)
0 4	Within the past year (6 months but less than 1 year ago)
0 5	Within the past 5 years (1 year but less than 5 years ago)
0 6	Within the past 10 years (5 years but less than 10 years ago)
0 7	10 years or more
7 7	Don’t know / Not sure
9 9	Refused

9.5 Do you currently use chewing tobacco, snuff, or snus every day, some days, or not at all?

Snus (rhymes with 'goose')

NOTE: Snus (Swedish for snuff) is a moist smokeless tobacco, usually sold in small pouches that are placed under the lip against the gum. (150)

1 Every day
2 Some days
3 Not at all

Do not read:

7 Don't know / Not sure
9 Refused

Section 10: Alcohol Consumption

10.1 During the past 30 days, how many days per week or per month did you have at least one drink of any alcoholic beverage such as beer, wine, a malt beverage, or liquor? (151-153)

1 _ _ Days per week
2 _ _ Days in past 30 days
8 8 8 No drinks in past 30 days **[Go to next section]**
7 7 7 Don't know / Not sure **[Go to next section]**
9 9 9 Refused **[Go to next section]**

10.2 One drink is equivalent to a 12-ounce beer, a 5-ounce glass of wine, or a drink with one shot of liquor. During the past 30 days, on the days when you drank, about how many drinks did you drink on the average? (154-155)

NOTE: A 40 ounce beer would count as 3 drinks, or a cocktail drink with 2 shots would count as 2 drinks.

_ _ Number of drinks
7 7 Don't know / Not sure
9 9 Refused

10.3 Considering all types of alcoholic beverages, how many times during the past 30 days did you have **X [CATI X = 5 for men, X = 4 for women]** or more drinks on an occasion? (156-157)

_ _ Number of times
8 8 None
7 7 Don't know / Not sure
9 9 Refused

10.4 During the past 30 days, what is the largest number of drinks you had on any occasion?
(158-159)

_ _ Number of drinks
7 7 Don't know / Not sure
9 9 Refused

Section 11: Immunization

11.1 Now I will ask you questions about the seasonal flu vaccine. There are two ways to get the seasonal flu vaccine, one is a shot in the arm and the other is a spray, mist, or drop in the nose called FluMist™. During the past 12 months, have you had either a seasonal flu shot or a seasonal flu vaccine that was sprayed in your nose?
(160)

READ IF NECESSARY:
A new flu shot came out in 2011 that injects vaccine into the skin with a very small needle. It is called Fluzone Intradermal vaccine. This is also considered a flu shot.

1 Yes
2 No **[Go to Q11.4]**
7 Don't know / Not sure **[Go to Q11.4]**
9 Refused **[Go to Q11.4]**

11.2 During what month and year did you receive your most recent flu shot injected into your arm or flu vaccine that was sprayed in your nose?
(161-166)

_ _ / _ _ _ _ Month / Year
7 7 / 7 7 7 7 Don't know / Not sure
9 9 / 9 9 9 9 Refused

11.3 At what kind of place did you get your last flu shot/vaccine?
(167-168)

0 1 A doctor's office or health maintenance organization (HMO)
0 2 A health department
0 3 Another type of clinic or health center (Example: a community health center)
0 4 A senior, recreation, or community center
0 5 A store (Examples: supermarket, drug store)
0 6 A hospital (Example: inpatient)
0 7 An emergency room
0 8 Workplace
0 9 Some other kind of place
1 0 Received vaccination in Canada/Mexico (Volunteered – Do not read)
1 1 A school
7 7 Don't know / Not sure **(*Probe:* "How would you describe the place where you went to get your most recent flu vaccine?"**

Do not read:

9 9 Refused

11.4 A pneumonia shot or pneumococcal vaccine is usually given only once or twice in a person's lifetime and is different from the flu shot. Have you ever had a pneumonia shot? (169)

1 Yes
2 No
7 Don't know / Not sure
9 Refused

Section 12: Falls

If respondent is 45 years or older continue, otherwise go to next section.

Next, I will ask about recent falls. By a fall, we mean when a person unintentionally comes to rest on the ground or another lower level.

12.1 In the past 12 months, how many times have you fallen? (170-171)

_ _	Number of times	**[76 = 76 or more]**
8 8	None	**[Go to next section]**
7 7	Don't know / Not sure	**[Go to next section]**
9 9	Refused	**[Go to next section]**

12.2 **[Fill in "Did this fall (from Q12.1) cause an injury?"]. If only one fall from Q12.1 and response is "Yes" (caused an injury); code 01. If response is "No," code 88.**

How many of these falls caused an injury? By an injury, we mean the fall caused you to limit your regular activities for at least a day or to go see a doctor. (172-173)

_ _	Number of falls	**[76 = 76 or more]**
8 8	None	
7 7	Don't know / Not sure	
9 9	Refused	

Section 13: Seatbelt Use

13.1 How often do you use seat belts when you drive or ride in a car? Would you say— (174)

Please read:

1 Always
2 Nearly always
3 Sometimes
4 Seldom
5 Never

Do not read:

7 Don't know / Not sure
8 Never drive or ride in a car
9 Refused

CATI note: If Q13.1 = 8 (Never drive or ride in a car), go to Section 15; otherwise continue.

Section 14: Drinking and Driving

CATI note: If Q10.1 = 888 (No drinks in the past 30 days); go to next section.

The next question is about drinking and driving.

14.1 During the past 30 days, how many times have you driven when you've had perhaps too much to drink?

(175-176)

_ _ Number of times
8 8 None
7 7 Don't know / Not sure
9 9 Refused

Section 15: Breast and Cervical Cancer Screening

CATI note: If respondent is male, go to the next section.

The next questions are about breast and cervical cancer.

15.1 A mammogram is an x-ray of each breast to look for breast cancer. Have you ever had a mammogram?

(177)

1 Yes
2 No **[Go to Q15.3]**
7 Don't know / Not sure **[Go to Q15.3]**
9 Refused **[Go to Q15.3]**

15.2 How long has it been since you had your last mammogram?

(178)

Read only if necessary:

1 Within the past year (anytime less than 12 months ago)
2 Within the past 2 years (1 year but less than 2 years ago)
3 Within the past 3 years (2 years but less than 3 years ago)
4 Within the past 5 years (3 years but less than 5 years ago)
5 5 or more years ago

Do not read:

7 Don't know / Not sure
9 Refused

15.3 A clinical breast exam is when a doctor, nurse, or other health professional feels the breasts for lumps. Have you ever had a clinical breast exam?

(179)

1 Yes
2 No **[Go to Q15.5]**
7 Don't know / Not sure **[Go to Q15.5]**
9 Refused **[Go to Q15.5]**

15.4 How long has it been since your last breast exam?

(180)

Read only if necessary:

1 Within the past year (anytime less than 12 months ago)
2 Within the past 2 years (1 year but less than 2 years ago)
3 Within the past 3 years (2 years but less than 3 years ago)
4 Within the past 5 years (3 years but less than 5 years ago)
5 5 or more years ago

Do not read:

7 Don't know / Not sure
9 Refused

15.5 A Pap test is a test for cancer of the cervix. Have you ever had a Pap test?

(181)

1 Yes
2 No **[Go to Q15.7]**
7 Don't know / Not sure **[Go to Q15.7]**
9 Refused **[Go to Q15.7]**

15.6 How long has it been since you had your last Pap test?

(182)

Read only if necessary:

1 Within the past year (anytime less than 12 months ago)
2 Within the past 2 years (1 year but less than 2 years ago)
3 Within the past 3 years (2 years but less than 3 years ago)
4 Within the past 5 years (3 years but less than 5 years ago)
5 5 or more years ago

Do not read:

7 Don't know / Not sure
9 Refused

CATI note: If response to Core Q7.21 = 1 (is pregnant); then go to next section.

15.7 Have you had a hysterectomy?

(183)

Read only if necessary: A hysterectomy is an operation to remove the uterus (womb).

1 Yes
2 No
7 Don't know / Not sure
9 Refused

Section 16: Prostate Cancer Screening

CATI note: If respondent is ≤39 years of age, or is female, go to next section.

Now, I will ask you some questions about prostate cancer screening.

16.1 A Prostate-Specific Antigen test, also called a PSA test, is a blood test used to check men for prostate cancer. Has a doctor, nurse, or other health professional EVER talked with you about the advantages of the PSA test?

(184)

1 Yes
2 No
7 Don't Know / Not sure
9 Refused

16.2 Has a doctor, nurse, or other health professional EVER talked with you about the disadvantages of the PSA test?

(185)

1 Yes
2 No
7 Don't Know / Not sure
9 Refused

16.3 Has a doctor, nurse, or other health professional EVER recommended that you have a PSA test?

(186)

1 Yes
2 No
7 Don't Know / Not sure
9 Refused

16.4 Have you EVER HAD a PSA test?

(187)

1 Yes
2 No **[Go to next section]**
7 Don't Know / Not sure **[Go to next section]**
9 Refused **[Go to next section]**

16.5 How long has it been since you had your last PSA test?

(188)

Read only if necessary:

1 Within the past year (anytime less than 12 months ago)
2 Within the past 2 years (1 year but less than 2 years)
3 Within the past 3 years (2 years but less than 3 years)
4 Within the past 5 years (3 years but less than 5 years)
5 5 or more years ago

Do not read:

7 Don't know / Not sure
9 Refused

16.6 What was the MAIN reason you had this PSA test – was it …?

(189)

Please read:

1 Part of a routine exam
2 Because of a prostate problem
3 Because of a family history of prostate cancer
4 Because you were told you had prostate cancer
5 Some other reason

Do Not Read:

7 Don't know / Not sure
9 Refused

Section 17: Colorectal Cancer Screening

CATI note: If respondent is ≤ 49 years of age, go to next section.

The next questions are about colorectal cancer screening.

17.1 A blood stool test is a test that may use a special kit at home to determine whether the stool contains blood. Have you ever had this test using a home kit?

(190)

1 Yes
2 No **[Go to Q17.3]**
7 Don't know / Not sure **[Go to Q17.3]**
9 Refused **[Go to Q17.3]**

17.2 How long has it been since you had your last blood stool test using a home kit?

(191)

Read only if necessary:

1 Within the past year (anytime less than 12 months ago)
2 Within the past 2 years (1 year but less than 2 years ago)
3 Within the past 3 years (2 years but less than 3 years ago)
4 Within the past 5 years (3 years but less than 5 years ago)
5 5 or more years ago

Do not read:

7 Don't know / Not sure
9 Refused

17.3 Sigmoidoscopy and colonoscopy are exams in which a tube is inserted in the rectum to view the colon for signs of cancer or other health problems. Have you ever had either of these exams?

(192)

1 Yes
2 No **[Go to next section]**
7 Don't know / Not sure **[Go to next section]**
9 Refused **[Go to next section]**

17.4 For a SIGMOIDOSCOPY, a flexible tube is inserted into the rectum to look for problems. A COLONOSCOPY is similar, but uses a longer tube, and you are usually given medication through a needle in your arm to make you sleepy and told to have someone else drive you home after the test. Was your MOST RECENT exam a sigmoidoscopy or a colonoscopy?

(193)

1 Sigmoidoscopy
2 Colonoscopy
7 Don't know / Not sure
9 Refused

17.5 How long has it been since you had your last sigmoidoscopy or colonoscopy?

(194)

Read only if necessary:

1 Within the past year (anytime less than 12 months ago)
2 Within the past 2 years (1 year but less than 2 years ago)
3 Within the past 3 years (2 years but less than 3 years ago)
4 Within the past 5 years (3 years but less than 5 years ago)
5 Within the past 10 years (5 years but less than 10 years ago)
6 10 or more years ago

Do not read:

7 Don't know / Not sure
9 Refused

Section 18: HIV/AIDS

The next few questions are about the national health problem of HIV, the virus that causes AIDS. Please remember that your answers are strictly confidential and that you do not have to answer every question if you do not want to. Although we will ask you about testing, we will not ask you about the results of any test you may have had.

18.1 Have you ever been tested for HIV? Do not count tests you may have had as part of a blood donation. Include testing fluid from your mouth.

(195)

1	Yes	
2	No	[Go to Q18.3]
7	Don't know / Not sure	[Go to Q18.3]
9	Refused	[Go to Q18.3]

18.2 Not including blood donations, in what month and year was your last HIV test?

(196-201)

NOTE: If response is before January 1985, code "Don't know."

CATI INSTRUCTION: If the respondent remembers the year but cannot remember the month, code the first two digits 77 and the last four digits for the year.

_ _ /_ _ _ _	Code month and year
7 7/ 7 7 7 7	Don't know / Not sure
9 9/ 9 9 9 9	Refused / Not sure

18.3 I'm going to read you a list. When I'm done, please tell me if any of the situations apply to you. You do not need to tell me which one.

- You have used intravenous drugs in the past year.
- You have been treated for a sexually transmitted or venereal disease in the past year.
- You have given or received money or drugs in exchange for sex in the past year.
- You had anal sex without a condom in the past year.

Do any of these situations apply to you?

(202)

1	Yes
2	No
7	Don't know / Not sure
9	Refused

Closing Statement or Transition to Modules and/or State-Added Questions

Closing statement

Please read:

That was my last question. Everyone's answers will be combined to help us provide information about the health practices of people in this state. Thank you very much for your time and cooperation.

Or

Transition to modules and/or state-added questions

Please read:

Finally, I have just a few questions left about some other health topics.

Optional Modules

Module 1: Pre-Diabetes

NOTE: Only asked of those not responding “Yes” (code = 1) to Core Q5.13 (Diabetes awareness question).

1. Have you had a test for high blood sugar or diabetes within the past three years?

(210)

1 Yes
2 No
7 Don’t know / Not sure
9 Refused

CATI NOTE: If Core Q5.13 = 4 (No, pre-diabetes or borderline diabetes); answer Q2 “Yes” (code = 1).

2. Have you ever been told by a doctor or other health professional that you have pre-diabetes or borderline diabetes?

If “Yes” and respondent is female, ask: “Was this only when you were pregnant?”

(211)

1 Yes
2 Yes, during pregnancy
3 No
7 Don’t know / Not sure
9 Refused

Module 2: Diabetes

To be asked following Core Q5.13; if response is "Yes" (code = 1)

1. How old were you when you were told you have diabetes?

(212-213)

_ _ Code age in years **[97 = 97 and older]**
9 8 Don’t know / Not sure
9 9 Refused

2. Are you now taking insulin?

(214)

1 Yes
2 No
9 Refused

3. About how often do you check your blood for glucose or sugar? Include times when checked by a family member or friend, but do NOT include times when checked by a health professional.

(215-217)

1 _ _ Times per day
2 _ _ Times per week
3 _ _ Times per month
4 _ _ Times per year
8 8 8 Never
7 7 7 Don't know / Not sure
9 9 9 Refused

4. About how often do you check your feet for any sores or irritations? Include times when checked by a family member or friend, but do NOT include times when checked by a health professional.

(218-220)

1 _ _ Times per day
2 _ _ Times per week
3 _ _ Times per month
4 _ _ Times per year
5 5 5 No feet
8 8 8 Never
7 7 7 Don't know / Not sure
9 9 9 Refused

5. About how many times in the past 12 months have you seen a doctor, nurse, or other health professional for your diabetes?

(221-222)

_ _ Number of times **[76 = 76 or more]**
8 8 None
7 7 Don't know / Not sure
9 9 Refused

6. A test for "A one C" measures the average level of blood sugar over the past three months. About how many times in the past 12 months has a doctor, nurse, or other health professional checked you for "A one C"?

(223-224)

_ _ Number of times **[76 = 76 or more]**
8 8 None
9 8 Never heard of "A one C" test
7 7 Don't know / Not sure
9 9 Refused

CATI note: If Q4 = 555 (No feet), go to Q8.

7. About how many times in the past 12 months has a health professional checked your feet for any sores or irritations?

(225-226)

_ _ Number of times **[76 = 76 or more]**
8 8 None
7 7 Don't know / Not sure
9 9 Refused

8. When was the last time you had an eye exam in which the pupils were dilated? This would have made you temporarily sensitive to bright light.

(227)

Read only if necessary:

1 Within the past month (anytime less than 1 month ago)
2 Within the past year (1 month but less than 12 months ago)
3 Within the past 2 years (1 year but less than 2 years ago)
4 2 or more years ago

Do not read:

7 Don't know / Not sure
1 Never
9 Refused

9. Has a doctor ever told you that diabetes has affected your eyes or that you had retinopathy?

(228)

1 Yes
2 No
7 Don't know / Not sure
9 Refused

10. Have you ever taken a course or class in how to manage your diabetes yourself?

(229)

1 Yes
2 No
7 Don't know / Not sure
9 Refused

Module 3: Healthy Days (Symptoms)

The next few questions are about health-related problems or symptoms.

1. During the past 30 days, for about how many days did pain make it hard for you to do your usual activities, such as self-care, work, or recreation?

(230-231)

_ _ Number of days
8 8 None
7 7 Don't know / Not sure
9 9 Refused

2. During the past 30 days, for about how many days have you felt sad, blue, or depressed?

(232-233)

_ _ Number of days
8 8 None
7 7 Don't know / Not sure
9 9 Refused

3. During the past 30 days, for about how many days have you felt worried, tense, or anxious?

(234-235)

_ _ Number of days
8 8 None
7 7 Don't know / Not sure
9 9 Refused

4. During the past 30 days, for about how many days have you felt very healthy and full of energy?

(236-237)

_ _ Number of days
8 8 None
7 7 Don't know / Not sure
9 9 Refused

Module 4: Visual Impairment and Access to Eye Care

CATI NOTE: If respondent is less than 40 years of age or Core Q5.12 = 3 (respondent is blind), go to next module.

Now I would like to ask you questions about your vision. These questions are for all respondents regardless of whether or not you wear glasses or contact lenses. If you wear glasses or contact lenses, answer questions as if you are wearing them.

1. How much difficulty, if any, do you have in recognizing a friend across the street? Would you say—

(238)

Please read:

1 No difficulty
2 A little difficulty
3 Moderate difficulty
4 Extreme difficulty
5 Unable to do because of eyesight

Or

6 Unable to do for other reasons

Do not read:

7 Don't know / Not sure
8 Not applicable (Blind) **[Go to next module]**
9 Refused

2. How much difficulty, if any, do you have reading print in newspapers, magazines, recipes, menus, or numbers on the telephone? Would you say—

(239)

Please read:

1 No difficulty
2 A little difficulty
3 Moderate difficulty
4 Extreme difficulty
5 Unable to do because of eyesight

Or

6 Unable to do for other reasons

Do not read:

7 Don't know / Not sure
8 Not applicable (Blind) **[Go to next module]**
9 Refused

3. When was the last time you had your eyes examined by any doctor or eye care provider? (240)

Read only if necessary:

1 Within the past month (anytime less than 1 month ago) **[Go to Q5]**
2 Within the past year (1 month but less than 12 months ago) **[Go to Q5]**
3 Within the past 2 years (1 year but less than 2 years ago)
4 2 or more years ago
5 Never

Do not read:

7 Don't know / Not sure
8 Not applicable (Blind) **[Go to next module]**
9 Refused

4. What is the main reason you have not visited an eye care professional in the past 12 months? (241-242)

Read only if necessary:

0 1 Cost/insurance
0 2 Do not have/know an eye doctor
0 3 Cannot get to the office/clinic (too far away, no transportation)
0 4 Could not get an appointment
0 5 No reason to go (no problem)
0 6 Have not thought of it
0 7 Other

Do not read:

7 7 Don't know / Not sure
0 8 Not Applicable (Blind) **[Go to next module]**
9 9 Refused

CATI note: Skip Q5, if any response to Module 2 (Diabetes) Q8.

5. When was the last time you had an eye exam in which the pupils were dilated? This would have made you temporarily sensitive to bright light. (243)

Read only if necessary:

1 Within the past month (anytime less than 1 month ago)
2 Within the past year (1 month but less than 12 months ago)
3 Within the past 2 years (1 year but less than 2 years ago)
4 2 or more years ago
5 Never

Do not read:

7 Don't know / Not sure
8 Not applicable (Blind) **[Go to next module]**
9 Refused

6. Do you have any kind of health insurance coverage for eye care?

(244)

1 Yes
2 No
7 Don't know / Not sure
8 Not applicable (Blind) **[Go to next module]**
9 Refused

7. Have you been told by an eye doctor or other health care professional that you NOW have cataracts?

(245)

1 Yes
2 No, I had them removed
3 No
7 Don't know / Not sure
8 Not applicable (Blind) **[Go to next module]**
9 Refused

8. Have you EVER been told by an eye doctor or other health care professional that you had glaucoma?

(246)

1 Yes
2 No
7 Don't know / Not sure
8 Not applicable (Blind) **[Go to next module]**
9 Refused

Please read:

Age-related Macular Degeneration (AMD) is a disease that affects the macula, the part of the eye that allows you to see fine detail.

NOTE: Age-related Macular Degeneration (Age-related Mak·yuh·luh Di·jen·uh·rey·shuh *n*)

9. Have you EVER been told by an eye doctor or other health care professional that you had age-related macular degeneration?

(247)

1 Yes
2 No
7 Don't know / Not sure
8 Not applicable (Blind)
9 Refused

Module 5: Sugar Sweetened Beverages and Menu Labeling

Now I would like to ask you some questions about sugary beverages.

Interviewer note: Please remind respondents to include regular soda that they mixed with alcohol.

1. During the past 30 days, how often did you drink regular soda or pop that contains sugar? Do not include diet soda or diet pop. (248-250)

1 _ _ Times per day
2 _ _ Times per week
3 _ _ Times per month

Do not read:

8 8 8 None
7 7 7 Don't know / Not sure
9 9 9 Refused

2. During the past 30 days, how often did you drink sweetened fruit drinks, such as Kool-aid, cranberry juice cocktail, and lemonade? Include fruit drinks you made at home and added sugar to. (251-253)

Interviewer note: Fruit drinks are sweetened beverages that often contain some fruit juice or flavoring. Do not include 100% fruit juice, sweet tea, coffee drinks, sports drinks, or energy drinks.

1 _ _ Times per day
2 _ _ Times per week
3 _ _ Times per month

Do not read:

8 8 8 None
7 7 7 Don't know / Not sure
9 9 9 Refused

3. The next question is about eating out at fast food and chain restaurants. When calorie information is available in the restaurant, how often does this information help you decide what to order?

(254-255)

Please read:

01 Always
02 Most of the time
03 About half the time
04 Sometimes
05 Never

Do not read:

06 Never noticed or never looked for calorie information
08 Usually cannot find calorie information
55 Do not eat at fast food or chain restaurants
77 Don't know / Not sure
99 Refused

Module 6: Excess Sun Exposure

1. In the past 12 months, how many times did you have a red OR painful sunburn that lasted a day or more?

(256)

8 Zero
1 One
2 Two
3 Three
4 Four
5 Five or more
7 Don't know / Not sure
9 Refused

Module 7: Inadequate Sleep

I would like to ask you a few questions about your sleep patterns.

1. During the past 30 days, for about how many days have you felt you did not get enough rest or sleep?

(257-258)

_ _ Number of days
8 8 None
7 7 Don't know / Not sure
9 9 Refused

2. On average, how many hours of sleep do you get in a 24-hour period? Think about the time you actually spend sleeping or napping, not just the amount of sleep you think you should get.

INTERVIEWER NOTE: Enter hours of sleep in whole numbers, rounding 30 minutes (1/2 hour) or more up to the next whole hour and dropping 29 or fewer minutes.

(259-260)

_ _ Number of hours [01-24]
7 7 Don't know / Not sure
9 9 Refused

3. Do you snore?

INTERVIEWER NOTE: If the respondent indicates that their spouse or someone told him/her that they snore, then the answer to the question is "Yes," the respondent snores.

(261)

1 Yes
2 No
7 Don't know / Not sure
9 Refused

4. During the past 30 days, for about how many days did you find yourself unintentionally falling asleep during the day?

(262-263)

_ _ Number of days [01-30]
8 8 None
7 7 Don't know / Not sure
9 9 Refused

5. During the past 30 days, have you ever nodded off or fallen asleep, even just for a brief moment, while driving?

(264)

1 Yes
2 No
3 Don't drive
4 Don't have license
7 Don't know / Not sure
9 Refused

Module 8: Fruits and Vegetables

These next questions are about the fruits and vegetables **you** ate or drank during the past 30 days. Please think about all forms of fruits and vegetables including cooked or raw, fresh, frozen or canned. Please think about all meals, snacks, and food consumed at home and away from home.

I will be asking how often **you** ate or drank each one: for example, once a day, twice a week, three times a month, and so forth.

INTERVIEWER NOTE: If respondent responds less than once per month, put "0" times per month. If respondent gives a number without a time frame, ask: "Was that per day, week, or month?"

1. During the past month, how many times per day, week or month did you drink 100% PURE fruit juices? Do not include fruit-flavored drinks with added sugar or fruit juice you made at home and added sugar to. Only include 100% juice.

(265-267)

1 _ _ Per day
2 _ _ Per week
3 _ _ Per month
5 5 5 Never
7 7 7 Don't know / Not sure
9 9 9 Refused

INTERVIEWER NOTE: Do not include fruit drinks with added sugar or other added sweeteners like Kool-aid, Hi-C, lemonade, cranberry cocktail, Tampico, Sunny Delight, Snapple, Fruitopia, Gatorade, Power-Ade, or yogurt drinks.

Do not include fruit juice drinks that provide 100% daily vitamin C but include added sugar.

Do not include vegetable juices such as tomato and V8 if respondent provides but include in "other vegetables" question 6.

DO include 100% pure juices including orange, mango, papaya, pineapple, apple, grape (white or red), or grapefruit. Only count cranberry juice if the respondent's perception is that it is 100% juice with no sugar or artificial sweetener added. 100% juice blends such as orange-pineapple, orange-tangerine, cranberry-grape are also acceptable as are fruit-vegetable 100% blends. 100% pure juice from concentrate (i.e., reconstituted) is counted.

2. During the past month, not counting juice, how many times per day, week, or month did you eat fruit? Count fresh, frozen, or canned fruit

(268-270)

1 _ _ Per day
2 _ _ Per week
3 _ _ Per month
5 5 5 Never
7 7 7 Don't know / Not sure
9 9 9 Refused

Read only if necessary: "Your best guess is fine. Include apples, bananas, applesauce, oranges, grape fruit, fruit salad, watermelon, cantaloupe or musk melon, papaya, lychees, star fruit, pomegranates, mangos, grapes, and berries such as blueberries and strawberries."

INTERVIEWER NOTE: Do not count fruit jam, jelly, or fruit preserves.

Do not include dried fruit in ready-to-eat cereals.

Do include dried raisins, cran-raisins if respondent tells you - ***but due to their small serving size they are not included in the prompt.***

Do include cut up fresh, frozen, or canned fruit added to yogurt, cereal, jello, and other meal items.

Include culturally and geographically appropriate fruits that are not mentioned (e.g. genip, soursop, sugar apple, figs, tamarind, bread fruit, sea grapes, carambola, longans, lychees, akee, rambutan, etc.).

3. During the past month, how many times per day, week, or month did you eat cooked or canned beans, such as refried, baked, black, garbanzo beans, beans in soup, soybeans, edamame, tofu or lentils. Do NOT include long green beans.

(271-273)

1 _ _ Per day
2 _ _ Per week
3 _ _ Per month
5 5 5 Never
7 7 7 Don't know / Not sure
9 9 9 Refused

Read only if necessary: "Include round or oval beans or peas such as navy, pinto, split peas, cow peas, hummus, lentils, soy beans and tofu. Do NOT include long green beans such as string beans, broad or winged beans, or pole beans."

INTERVIEWER NOTE: Include soybeans also called edamame, TOFU (BEAN CURD MADE FROM SOYBEANS), kidney, pinto, hummus, lentils, black, black-eyed peas, cow peas, lima beans and white beans.

Include bean burgers including garden burgers and veggie burgers.

Include falafel and tempeh.

4. During the past month, how many times per day, week, or month did you eat dark green vegetables for example broccoli or dark leafy greens including romaine, chard, collard greens or spinach?

(274-276)

1 _ _ Per day
2 _ _ Per week
3 _ _ Per month
5 5 5 Never
7 7 7 Don't know / Not sure
9 9 9 Refused

INTERVIEWER NOTE: Each time a vegetable is eaten it counts as one time.

INTERVIEWER NOTE: Include all raw leafy green salads including spinach, mesclun, romaine lettuce, bok choy, dark green leafy lettuce, dandelions, komatsuna, watercress, and arugula.

Do not include iceberg (head) lettuce if specifically told type of lettuce. Include all cooked greens including kale, collard greens, choys, turnip greens, mustard greens.

5. During the past month, how many times per day, week, or month did you eat orange-colored vegetables such as sweet potatoes, pumpkin, winter squash, or carrots?
(277-279)

1 _ _ Per day
2 _ _ Per week
3 _ _ Per month
5 5 5 Never
7 7 7 Don't know / Not sure
9 9 9 Refused

Read only if needed: "Winter squash have hard, thick skins and deep yellow to orange flesh. They include acorn, buttercup, and spaghetti squash."

FOR INTERVIEWER: Include all forms of carrots including long or baby-cut.

Include carrot-slaw (e.g. shredded carrots with or without other vegetables or fruit).

Include all forms of sweet potatoes including baked, mashed, casserole, pie, or sweet potatoes fries.

Include all hard-winter squash varieties including acorn, autumn cup, banana, butternut, buttercup, delicate, hubbard, kabocha (Also known as an Ebisu, Delica, Hoka, Hokkaido, or Japanese Pumpkin; blue kuri), and spaghetti squash. Include all forms including soup.

Include pumpkin, including pumpkin soup and pie. Do not include pumpkin bars, cake, bread or other grain-based dessert-type food containing pumpkin (i.e. similar to banana bars, zucchini bars we do not include).

6. Not counting what you just told me about, during the past month, about how many times per day, week, or month did you eat OTHER vegetables? Examples of other vegetables include tomatoes, tomato juice or V-8 juice, corn, eggplant, peas, lettuce, cabbage, and white potatoes that are not fried such as baked or mashed potatoes.
(280-282)

1 _ _ Per day
2 _ _ Per week
3 _ _ Per month
5 5 5 Never
7 7 7 Don't know / Not sure
9 9 9 Refused

Read only if needed: "Do not count vegetables you have already counted and do not include fried potatoes."

INTERVIEWER NOTE: Include corn, peas, tomatoes, okra, beets, cauliflower, bean sprouts, avocado, cucumber, onions, peppers (red, green, yellow, orange); all cabbage including American-style cole-slaw; mushrooms, snow peas, snap peas, broad beans, string, wax-, or pole-beans.

Include any form of the vegetable (raw, cooked, canned, or frozen).

Do not include products consumed usually as condiments including ketchup, catsup, salsa, chutney, relish.

Do include tomato juice if respondent did not count in fruit juice.

Include culturally and geographically appropriate vegetables that are not mentioned (e.g. daikon, jicama, oriental cucumber, etc.).

Do not include rice or other grains.

Module 9: Adult Asthma History

CATI NOTE: If "Yes" to Core Q5.4; continue. Otherwise, go to next module.

Previously you said you were told by a doctor, nurse, or other health professional that you had asthma.

1. How old were you when you were first told by a doctor, nurse, or other health professional that you had asthma?

(283-284)

_ _ Age in years 11 or older **[96 = 96 and older]**
9 7 Age 10 or younger
9 8 Don’t know / Not sure
9 9 Refused

CATI NOTE: If "Yes" to Core Q5.5, continue. Otherwise, go to next module.

2. During the past 12 months, have you had an episode of asthma or an asthma attack?

(285)

1 Yes
2 No **[Go to Q5]**
7 Don’t know / Not sure **[Go to Q5]**
9 Refused **[Go to Q5]**

3. During the past 12 months, how many times did you visit an emergency room or urgent care center because of your asthma?

(286-287)

_ _ Number of visits **[87 = 87 or more]**
8 8 None
9 8 Don’t know / Not sure
9 9 Refused

4. **[If one or more visits to Q3, fill in “Besides those emergency room or urgent care center visits,”]** During the past 12 months, how many times did you see a doctor, nurse or other health professional for urgent treatment of worsening asthma symptoms?

(288-289)

_ _ Number of visits **[87 = 87 or more]**
8 8 None
9 8 Don’t know / Not sure
9 9 Refused

5. During the past 12 months, how many times did you see a doctor, nurse, or other health professional for a routine checkup for your asthma?

(290-291)

_ _ Number of visits **[87 = 87 or more]**
8 8 None
9 8 Don't know / Not sure
9 9 Refused

6. During the past 12 months, how many days were you unable to work or carry out your usual activities because of your asthma?

(292-294)

_ _ _ Number of days
8 8 8 None
7 7 7 Don't know / Not sure
9 9 9 Refused

7. Symptoms of asthma include cough, wheezing, shortness of breath, chest tightness and phlegm production when you don't have a cold or respiratory infection. During the past 30 days, how often did you have any symptoms of asthma? Would you say —

(295)

NOTE: Phlegm ('flem')

Please read:

8 Not at any time **[Go to Q9]**
1 Less than once a week
2 Once or twice a week
3 More than 2 times a week, but not every day
4 Every day, but not all the time

Or

5 Every day, all the time

Do not read:

7 Don't know / Not sure
9 Refused

8. During the past 30 days, how many days did symptoms of asthma make it difficult for you to stay asleep? Would you say —

(296)

Please read:

8 None
1 One or two
2 Three to four
3 Five
4 Six to ten

Or

5 More than ten

Do not read:

7 Don't know / Not sure
9 Refused

9. During the past 30 days, how many days did you take a prescription asthma medication to PREVENT an asthma attack from occurring?

(297)

Please read:

8 Never
1 1 to 14 days
2 15 to 24 days
3 25 to 30 days

Do not read:

7 Don't know / Not sure
9 Refused

10. During the past 30 days, how often did you use a prescription asthma inhaler DURING AN ASTHMA ATTACK to stop it?

(298)

INTERVIEWER INSTRUCTION: How often (number of times) does NOT equal number of puffs. Two to three puffs are usually taken each time the inhaler is used.

Read only if necessary:

8 Never (include no attack in past 30 days)
1 1 to 4 times (in the past 30 days)
2 5 to 14 times (in the past 30 days)
3 15 to 29 times (in the past 30 days)
4 30 to 59 times (in the past 30 days)
5 60 to 99 times (in the past 30 days)
6 100 or more times (in the past 30 days)

Do not read:

7 Don't know / Not sure
9 Refused

Module 10: High Risk/Health Care Worker

The next few questions ask about health care work and chronic illness.

1. Do you currently volunteer or work in a hospital, medical clinic, doctor's office, dentist's office, nursing home or some other health-care facility? This includes part-time and unpaid work in a health care facility as well as professional nursing care provided in the home.

(299)

INTERVIEWER NOTE: If necessary say: "This includes non-health care professionals, such as administrative staff, who work in a health-care facility."

1	Yes	
2	No	**[Go to Q3]**
7	Don't know / Not sure	**[Go to Q3]**
9	Refused	**[Go to Q3]**

2. Do you provide direct patient care as part of your routine work? By direct patient care we mean physical or hands-on contact with patients.

(300)

1 Yes
2 No
7 Don't know / Not sure ***(Probe by repeating question)***
9 Refused

3. Has a doctor, nurse, or other health professional ever said that you have…

<u>**Read all items listed below before waiting for an answer:**</u>

Lung problems, other than asthma
Kidney problems
Anemia, including Sickle Cell

Or

A weakened immune system caused by a chronic illness or by medicines taken for a chronic illness?

(301)

[See Attached Health Problems List, if necessary]

1	Yes	
2	No	**[Go to next module]**
7	Don't know / Not sure	**[Go to next module]**
9	Refused	**[Go to next module]**

4. Do you still have (this/any of these) problem(s)?

(302)

1 Yes
2 No
7 Don't know / Not sure
9 Refused

Module 11: Shingles (Zostavax or ZOS)

CATI note: If respondent is ≤ 49 years of age, go to next module.

The next question is about the Shingles vaccine.

1. Shingles is caused by the chicken pox virus. It is an outbreak of rash or blisters on the skin that may be associated with severe pain. A vaccine for shingles has been available since May 2006; it is called Zostavax®, the zoster vaccine, or the shingles vaccine. Have you had this vaccine?

(303)

1 Yes
2 No
7 Don't know / Not sure
9 Refused

Module 12: Tetanus Diphtheria (Adults)

Next, I will ask you about the tetanus diphtheria vaccination.

1. Have you received a tetanus shot in the past 10 years?

(304)

1 Yes
2 No **[Go to next module]**
7 Don't know / Not sure **[Go to next module]**
9 Refused **[Go to next module]**

2. Was your most recent tetanus shot given in 2005 or later?

(305)

1 Yes
2 No **[Go to next module]**
7 Don't know / Not sure
9 Refused

3. There are currently two types of tetanus shots available for adults. One contains the tetanus diphtheria vaccine. The other type contains tetanus diphtheria and pertussis or whooping cough vaccine. Did your doctor say your recent tetanus shot included the pertussis or whooping cough vaccine?

(306)

1 Yes (included pertussis)
2 No (did not include pertussis)
7 Don't know / Not sure
9 Refused

Module 13: Adult Human Papilloma Virus (HPV)

CATI note: To be asked of respondents between the ages of 18 and 49 years; otherwise, go to next module.

NOTE: Human Papilloma Virus (Human Pap·uh·loh·muh Virus); Gardasil (Gar·duh· seel); Cervarix (Sir·var· icks)

1. A vaccine to prevent the human papilloma virus or HPV infection is available and is called the cervical cancer or genital warts vaccine, HPV shot, **[Fill: if female "GARDASIL or CERVARIX"; if male " or GARDASIL"].** Have you EVER had an HPV vaccination?

(307)

1	Yes	
2	No	**[Go to next module]**
3	Doctor refused when asked	**[Go to next module]**
7	Don't know / Not sure	**[Go to next module]**
9	Refused	**[Go to next module]**

2. How many HPV shots did you receive?

(308-309)

_ _ Number of shots
0 3 All shots
7 7 Don't know / Not sure
9 9 Refused

Module 14: Prostate Cancer Screening Decision Making Module

CATI Note: If Q16.4 = 1 (has had a PSA test) continue, else go to next module.

1. Which one of the following best describes the decision to have the PSA test done? (310)

Please read:

1 You made the decision alone **[Go to next module]**
2 Your doctor, nurse, or health care provider made the decision alone **[Go to next module]**
3 You and one or more other persons made the decision together
4 You don't remember how the decision was made **[Go to next module]**

Do not read:

9 Refused

2. Who made the decision with you? (Mark all that apply) (311-315)

1 Doctor/nurse /health care provider
2 Spouse/significant other
3 Other family member
4 Friend/non-relative

8 No additional choices
7 Don't know / Not sure
9 Refused

Module 15: Cancer Survivorship

CATI note: If Core Q5.6 or Q5.7 = 1 (Yes) or Q16.6 = 4 (Because you were told you had prostate cancer) continue, else go to next module.

You've told us that you have had cancer. I would like to ask you a few more questions about your cancer.

1. How many different types of cancer have you had? (316)

1 Only one
2 Two
3 Three or more
7 Don't know / Not sure **[Go to next module]**
9 Refused **[Go to next module]**

2. At what age were you told that you had cancer?

(317-318)

_ _ Code age in years **[97 = 97 and older]**
9 8 Don't know / Not sure
9 9 Refused

CATI NOTE: If Q1= 2 (Two) or 3 (Three or more), ask: "At what age were you first diagnosed with cancer?"

INTERVIEWER NOTE: This question refers to the first time they were told about their first cancer.

CATI NOTE: If Core Q5.6 = 1 (Yes) and Q1 = 1 (Only one): ask "Was it "Melanoma" or "other skin cancer"? **then code 21 if "Melanoma" or 22 if "other skin cancer"**

CATI NOTE: If Core Q16.6 = 4 (Because you were told you had Prostate Cancer) and Q1 = 1 (Only one) then code 19.

3. What type of cancer was it?

(319-320)

If Q1 = 2 (Two) or 3 (Three or more), ask: "With your most recent diagnoses of cancer, what type of cancer was it?"

INTERVIEWER NOTE: Please read list only if respondent needs prompting for cancer type (i.e., name of cancer) [1-30]:

Breast
0 1 Breast cancer

Female reproductive (Gynecologic)
0 2 Cervical cancer (cancer of the cervix)
0 3 Endometrial cancer (cancer of the uterus)
0 4 Ovarian cancer (cancer of the ovary)

Head/Neck
0 5 Head and neck cancer
0 6 Oral cancer
0 7 Pharyngeal (throat) cancer
0 8 Thyroid
0 9 Larynx

Gastrointestinal
1 0 Colon (intestine) cancer
1 1 Esophageal (esophagus)
1 2 Liver cancer
1 3 Pancreatic (pancreas) cancer
1 4 Rectal (rectum) cancer
1 5 Stomach

Leukemia/Lymphoma (lymph nodes and bone marrow)
1 6 Hodgkin's Lymphoma (Hodgkin's disease)
1 7 Leukemia (blood) cancer
1 8 Non-Hodgkin's Lymphoma

Male reproductive
1 9 Prostate cancer
2 0 Testicular cancer

Skin
2 1 Melanoma
2 2 Other skin cancer

Thoracic
2 3 Heart
2 4 Lung

Urinary cancer:
2 5 Bladder cancer
2 6 Renal (kidney) cancer

Others
2 7 Bone
2 8 Brain
2 9 Neuroblastoma
3 0 Other

Do not read:

7 7 Don't know / Not sure
9 9 Refused

4. Are you currently receiving treatment for cancer? By treatment, we mean surgery, radiation therapy, chemotherapy, or chemotherapy pills.

(321)

1	Yes	**[Go to next module]**
2	No, I've completed treatment	
3	No, I've refused treatment	**[Go to next module]**
4	No, I haven't started treatment	**[Go to next module]**
7	Don't know / Not sure	**[Go to next module]**
9	Refused	**[Go to next module]**

5. What type of doctor provides the majority of your health care?

(322-323)

INTERVIEWER NOTE: If the respondent requests clarification of this question, say: “We want to know which type of doctor you see most often for illness or regular health care (Examples: annual exams and/or physicals, treatment of colds, etc.).”

Please read [1-10]:

0 1 Cancer Surgeon
0 2 Family Practitioner
0 3 General Surgeon
0 4 Gynecologic Oncologist
0 5 General Practitioner, Internist
0 6 Plastic Surgeon, Reconstructive Surgeon
0 7 Medical Oncologist
0 8 Radiation Oncologist
0 9 Urologist
1 0 Other

Do not read:

7 7 Don’t know / Not sure
9 9 Refused

6. Did any doctor, nurse, or other health professional EVER give you a written summary of all the cancer treatments that you received?

(324)

Read only if necessary: “By ‘other healthcare professional’, we mean a nurse practitioner, a physician’s assistant, social worker, or some other licensed professional.”

1 Yes
2 No
7 Don’t know / Not sure
9 Refused

7. Have you EVER received instructions from a doctor, nurse, or other health professional about *where* you should return or *who* you should see for routine cancer check-ups after completing your treatment for cancer?

(325)

1 Yes
2 No **[Go to Q9]**
7 Don’t know / Not sure **[Go to Q9]**
9 Refused **[Go to Q9]**

8. Were these instructions written down or printed on paper for you? (326)

1 Yes
2 No
7 Don’t know / Not sure
9 Refused

9. With your most recent diagnosis of cancer, did you have health insurance that paid for all or part of your cancer treatment?
(327)

1 Yes
2 No
7 Don’t know / Not sure
9 Refused

INTERVIEWER NOTE: “Health insurance” also includes Medicare, Medicaid, or other types of state health programs.

10. Were you EVER denied health insurance or life insurance coverage because of your cancer?
(328)

1 Yes
2 No
7 Don’t know / Not sure
9 Refused

11. Did you participate in a clinical trial as part of your cancer treatment?
(329)

1 Yes
2 No
7 Don’t know / Not sure
9 Refused

12. Do you currently have physical pain caused by your cancer or cancer treatment?
(330)

1 Yes
2 No **[Go to next module]**
7 Don’t know / Not sure **[Go to next module]**
9 Refused **[Go to next module]**

13. Is your pain currently under control?
(331)

Please read:

1 Yes, with medication (or treatment)
2 Yes, without medication (or treatment)
3 No, with medication (or treatment)

4 No, without medication (or treatment)

Do not read:

7 Don't know / Not sure
9 Refused

Module 16: Reactions to Race

Earlier I asked you to self-identify your race. Now I will ask you how other people identify you and treat you.

1. How do other people usually classify you in this country? Would you say: White, Black or African American, Hispanic or Latino, Asian, Native Hawaiian or Other Pacific Islander, American Indian or Alaska Native, or some other group?

(332)

1 White
2 Black or African American
3 Hispanic or Latino
4 Asian
5 Native Hawaiian or Other Pacific Islander
6 American Indian or Alaska Native
8 Some other group (please specify) ________________________
7 Don't know / Not sure
9 Refused

INTERVIEWER NOTE: If the respondent requests clarification of this question, say: "We want to know how OTHER people usually classify you in this country, which might be different from how you classify yourself."

2. How often do you think about your race? Would you say never, once a year, once a month, once a week, once a day, once an hour, or constantly?

(333)

1 Never
2 Once a year
3 Once a month
4 Once a week
5 Once a day
6 Once an hour
8 Constantly
7 Don't know / Not sure
9 Refused

INTERVIEWER INSTRUCTION: The responses can be interpreted as meaning "at least" the indicated time frequency. If a respondent cannot decide between two categories, check the response for the lower frequency. For example, if a respondent says that they think about their race between once a week and once a month, check "once a month" as the response.

[CATI skip pattern: This question should only be asked of those who are "employed for wages," "self-employed," or "out of work for less than one year."]

3. Within the past 12 months at work, do you feel you were treated worse than, the same as, or better than people of other races?

(334)

1 Worse than other races
2 The same as other races
3 Better than other races

Do not read:

4 Worse than some races, better than others
5 Only encountered people of the same race
7 Don't know / Not sure
9 Refused

4. Within the past 12 months, when seeking health care, do you feel your experiences were worse than, the same as, or better than for people of other races?

(335)

1 Worse than other races
2 The same as other races
3 Better than other races

Do not read:

4 Worse than some races, better than others
5 Only encountered people of the same race
6 No health care in past 12 months
7 Don't know / Not sure
9 Refused

INTERVIEWER NOTE: If the respondent indicates that they do not know about other people's experiences when seeking health care, say: "This question is asking about your perceptions when seeking health care. It does not require specific knowledge about other people's experiences."

5. Within the past 30 days, have you experienced any physical symptoms, for example, a headache, an upset stomach, tensing of your muscles, or a pounding heart, as a result of how you were treated based on your race?

(336)

1 Yes
2 No
7 Don't know / Not sure
9 Refused

6. Within the past 30 days, have you felt emotionally upset, for example angry, sad, or frustrated, as a result of how you were treated based on your race?

(337)

1 Yes
2 No
7 Don't know / Not sure
9 Refused

Now, I am going to ask you some questions about how you have been feeling lately.

1. About how often during the past 30 days did you feel **nervous** — would you say **all** of the time, **most** of the time, **some** of the time, **a little** of the time, or **none** of the time?

(338)

1 All
2 Most
3 Some
4 A little
5 None
7 Don't know / Not sure
9 Refused

2. During the past 30 days, about how often did you feel **hopeless** — **all** of the time, **most** of the time, **some** of the time, **a little** of the time, or **none** of the time?

(339)

1 All
2 Most
3 Some
4 A little
5 None
7 Don't know / Not sure
9 Refused

3. During the past 30 days, about how often did you feel **restless** or **fidgety**?

[If necessary: all, most, some, a little, or none of the time?]

(340)

1 All
2 Most
3 Some
4 A little
5 None
7 Don't know / Not sure
9 Refused

4. During the past 30 days**,** about how often did you feel **so depressed** that nothing could cheer you up?

[If necessary: all, most, some, a little, or none of the time?]

(341)

1 All
2 Most
3 Some
4 A little
5 None
7 Don't know / Not sure
9 Refused

5. During the past 30 days, about how often did you feel that **everything was an effort**?

Note: If respondent asks what does "everything was an effort" means; say, "Whatever it means to you"

[If necessary: all, most, some, a little, or none of the time?]

(342)

1 All
2 Most
3 Some
4 A little
5 None
7 Don't know / Not sure
9 Refused

6. During the past 30 days, about how often did you feel **worthless**?

[If necessary: all, most, some, a little, or none of the time?]

(343)

1 All
2 Most
3 Some
4 A little
5 None
7 Don't know / Not sure
9 Refused

7. During the past 30 days, for about how many days did a mental health condition or emotional problem **keep you from doing** your work or other usual activities?

(344-345)

_ _ Number of days
8 8 None
7 7 Don't know / Not sure
9 9 Refused

INTERVIEWER NOTE: If asked, **"usual activities"** includes housework, self-care, care giving, volunteer work, attending school, studies, or recreation.

8. Are you now taking medicine or receiving treatment from a doctor or other health professional for any type of mental health condition or emotional problem?

(346)

1 Yes
2 No
7 Don't know / Not sure
9 Refused

These next questions ask about peoples' attitudes toward mental illness and its treatment.

9. Treatment can help people with mental illness lead normal lives. Do you –**agree** slightly or strongly, or **disagree** slightly or strongly?

(347)

Read only if necessary:

1 Agree strongly
2 Agree slightly
3 Neither agree nor disagree
4 Disagree slightly
5 Disagree strongly

Do not read:

7 Don't know / Not sure
9 Refused

10. People are generally caring and sympathetic to people with mental illness. Do you – **agree** slightly or strongly, or **disagree** slightly or strongly?

(348)

Read only if necessary:

1 Agree strongly
2 Agree slightly
3 Neither agree nor disagree
4 Disagree slightly
5 Disagree strongly

Do not read:

7 Don't know / Not sure
9 Refused

INTERVIEWER NOTE: If asked for the purpose of Q9 or Q10: say: "answers to these questions will be used by health planners to help understand public attitudes about mental illness and its treatment and to help guide health education programs".

Module 18: Social Context

Now, I am going to ask you about several factors that can affect a person's health.

If Core Q7.21 = 1 or 2 (own or rent) continue, else go to Q2.

1. How often in the past 12 months would you say you were worried or stressed about having enough money to pay your rent/mortgage? Would you say you were worried or stressed---

(349)

Please read:

1 Always
2 Usually
3 Sometimes
4 Rarely
5 Never

Do not read:

8 Not applicable
7 Don't know / Not sure
9 Refused

2. How often in the past 12 months would you say you were worried or stressed about having enough money to buy nutritious meals? Would you say you were worried or stressed---

(350)

Please read:

1 Always
2 Usually
3 Sometimes
4 Rarely
5 Never

Do not read:

8 Not applicable
7 Don't know / Not sure
9 Refused

If Core Q7.9 = 1 (Employed for wages) or 2 (Self-employed), go to Q3 and Q4.

If Core Q7.9 = 3 (Out of work for more than 1 year), 4 (Out of work for less than 1 year), or 7 (Retired), go to Q5 and Q6.

If Core Q7.9 = 5 (A homemaker), 6 (A student), or 8 (Unable to work), go to Q6.

3. At your main job or business, how are you generally paid for the work you do. Are you:

(351)

1 Paid by salary
2 Paid by the hour
3 Paid by the job/task (e.g. commission, piecework)
4 Paid some other way
7 Don't know / Not sure
9 Refused

INTERVIEWER NOTE: If paid in multiple ways at their main job, select option 4 (Paid some other way).

4. About how many hours do you work per week at all of your jobs and businesses combined?

(352-353)

_ _	Hours (01-96 or more)	**[Go to next module]**
9 7	Don't know / Not sure	**[Go to next module]**
9 8	Does not work	**[Go to next module]**
9 9	Refused	**[Go to next module]**

5. Thinking about the last time you worked, at your main job or business, how were you generally paid for the work you did? Were you:

(354)

1 Paid by salary
2 Paid by the hour
3 Paid by the job/task (e.g. commission, piecework)
4 Paid some other way
7 Don't know / Not sure
9 Refused

6. Thinking about the last time you worked, about how many hours did you work per week at all of your jobs and businesses combined?

(355-356)

_ _ Hours (01-96 or more)
9 7 Don't know / Not sure
9 8 Does not work
9 9 Refused

Module 19: General Preparedness

The next series of questions asks about how prepared you are for a large-scale disaster or emergency. By large-scale disaster or emergency we mean any event that leaves you isolated in your home ***or*** displaces you from your home for at least 3 days. This might include natural disasters such as hurricanes, tornados, floods, and ice storms, or man-made disasters such as explosions, terrorist events, or blackouts.

1. How well prepared do you feel your household is to handle a large-scale disaster or emergency? Would you say…

(357)

Please read:

1 Well prepared
2 Somewhat prepared
3 Not prepared at all

Do not read:

7 Don't know / Not sure
9 Refused

2. Does your household have a 3-day supply of water for everyone who lives there? A 3-day supply of water is 1 gallon of water per person per day.

(358)

1 Yes
2 No
7 Don't know / Not sure
9 Refused

3. Does your household have a 3-day supply of nonperishable food for everyone who lives there? By nonperishable we mean food that does not require refrigeration or cooking.

(359)

1 Yes
2 No
7 Don't know / Not sure
9 Refused

4. Does your household have a 3-day supply of prescription medication for each person who takes prescribed medicines?

(360)

1 Yes
2 No
3 No one in household requires prescribed medicine
7 Don't know / Not sure
9 Refused

5. Does your household have a working battery operated radio and working batteries for your use if the electricity is out?

(361)

1 Yes
2 No
7 Don't know / Not sure
9 Refused

6. Does your household have a working flashlight and working batteries for your use if the electricity is out?

(362)

1 Yes
2 No
7 Don't know / Not sure
9 Refused

7. In a large-scale disaster or emergency, what would be your main method or way of communicating with relatives and friends?

(363)

Read only if necessary:

1 Regular home telephones
2 Cell phones
3 Email
4 Pager
5 2-way radios
6 Other

Do not read:

7 Don't know / Not sure
9 Refused

8. What would be your main method or way of getting information from authorities in a large-scale disaster or emergency?

(364)

Read only if necessary:

1 Television
2 Radio
3 Internet
4 Print media
5 Neighbors
6 Other

Do not read:

7 Don't know / Not sure
9 Refused

9. Does your household have a written disaster evacuation plan for how you will leave your home, in case of a large-scale disaster or emergency that requires evacuation?

(365)

1 Yes
2 No
7 Don't know / Not sure
9 Refused

10. If public authorities announced a mandatory evacuation from your community due to a large-scale disaster or emergency, would you evacuate?

(366)

1 Yes **[Go to next module]**
2 No
7 Don't know / Not sure
9 Refused

11. What would be the main reason you might not evacuate if asked to do so?

(367-368)

Read only if necessary:

0 1 Lack of transportation
0 2 Lack of trust in public officials
0 3 Concern about leaving property behind
0 4 Concern about personal safety
0 5 Concern about family safety
0 6 Concern about leaving pets
0 7 Concern about traffic jams and inability to get out
0 8 Health problems (could not be moved)
0 9 Other

Do not read:

7 7 Don't know / Not sure
9 9 Refused

Module 20: Veteran's Health

CATI NOTE: If Core Q7.5 = 1 (Yes) continue, else go to next module.

The next questions relate to veteran's health.

1. Did you ever serve in a combat or war zone?

(369)

1 Yes
2 No
7 Don't know / Not sure
9 Refused

2. Has a doctor or other health professional ever told you that you have depression, anxiety, or post traumatic stress disorder (PTSD)?

(370)

1 Yes
2 No
7 Don't know / Not sure
9 Refused

3. A traumatic brain injury may result from a violent blow to the head or when an object pierces the skull and enters the brain tissue. Has a doctor or other health professional ever told you that you have suffered a traumatic brain injury (TBI)?

(371)

1 Yes
2 No
7 Don't know / Not sure
9 Refused

4. In the past 12 months, did you receive any psychological or psychiatric counseling or treatment?

(372)

Please read:

1 Yes, from a VA facility
2 Yes, from a non-VA facility
3 Yes, from both VA and non-VA facilities
4 No

Do not read:

7 Don't know / Not sure
9 Refused

The next few questions are a sensitive topic and some people may feel uncomfortable with these questions. At the end of this section, I will give you a phone number for an organization that can provide information and referral for these issues. Please keep in mind that you can ask me to skip any question you do not want to answer.

5. Has there been a time in the past 12 months when you thought of taking your own life?

(373)

1	Yes	
2	No	**[Go to next module]**
7	Don't know / Not sure	**[Go to next module]**
9	Refused	**[Go to next module]**

6. During the past 12 months, did you attempt to commit suicide? Would you say---

(374)

Please read:

1 Yes, but did not require treatment
2 Yes, was treated at a VA facility
3 Yes, was treated at a non-VA facility
4 No

Do not read:

7 Don't know / Not sure
9 Refused

As I mentioned, I would give you a phone number for an organization that can provide information and referral for these issues. You can dial the National Crisis line at 1-800-273-TALK (8255). You can also speak directly to your doctor or health provider.

Module 21: Chronic Obstructive Pulmonary Disease (COPD)

CATI NOTE: If core Q5.8 = 1 (Yes) then continue, else go to next module.

Earlier you said that you had been diagnosed with Chronic Obstructive Pulmonary Disease or COPD.

1. Have you ever been given a breathing test to diagnose your COPD, chronic bronchitis, or emphysema?

(375)

1 Yes
2 No
7 Don't know / Not sure
9 Refused

2. Would you say that shortness of breath affects the quality of your life?

(376)

1 Yes
2 No
7 Don't know / Not sure
9 Refused

3. Other than a routine visit, have you had to see a doctor in the past 12 months for symptoms related to shortness of breath, bronchitis, or other COPD, or emphysema flare?

(377)

1 Yes
2 No
7 Don't know / Not sure
9 Refused

4. Did you have to visit an emergency room or be admitted to the hospital in the past 12 months because of your COPD, chronic bronchitis, or emphysema?

(378)

1 Yes
2 No
7 Don't know / Not sure
9 Refused

5. How many different medications do you currently take each day to help with your COPD, chronic bronchitis, or emphysema?

(379-380)

_ _ Number (01-76)
7 7 Don't know / Not sure
8 8 None
9 9 Refused

Module 22: Adverse Childhood Experience

I'd like to ask you some questions about events that happened during your childhood. This information will allow us to better understand problems that may occur early in life, and may help others in the future. This is a sensitive topic and some people may feel uncomfortable with these questions. At the end of this section, I will give you a phone number for an organization that can provide information and referral for these issues. Please keep in mind that you can ask me to skip any question you do not want to answer.

All questions refer to the time period before you were 18 years of age. Now, looking back before you were 18 years of age—

1. Did you live with anyone who was depressed, mentally ill, or suicidal? (381)

1 Yes
2 No
7 Don't know / Not sure
9 Refused

2. Did you live with anyone who was a problem drinker or alcoholic? (382)

1 Yes
2 No
7 Don't know / Not sure
9 Refused

3. Did you live with anyone who used illegal street drugs or who abused prescription medications? (383)

1 Yes
2 No
7 Don't know / Not sure
9 Refused

4. Did you live with anyone who served time or was sentenced to serve time in a prison, jail, or other correctional facility? (384)

1 Yes
2 No
7 Don't know / Not sure
9 Refused

5. Were your parents separated or divorced? (385)

1 Yes
2 No
8 Parents not married
7 Don't know / Not sure
9 Refused

6. How often did your parents or adults in your home ever slap, hit, kick, punch, or beat each other up?

(386)

1 Never
2 Once
3 More than once

Do not read:

7 Don't know / Not sure
9 Refused

7. Before age 18, how often did a parent or adult in your home ever hit, beat, kick, or physically hurt you in any way? Do not include spanking. Would you say---

(387)

1 Never
2 Once
3 More than once

Do not read:

7 Don't know / Not sure
9 Refused

8. How often did a parent or adult in your home ever swear at you, insult you, or put you down?

(388)

1 Never
2 Once
3 More than once

Do not read:

7 Don't know / Not sure
9 Refused

9. How often did anyone at least 5 years older than you or an adult, ever touch you sexually?

(389)

1 Never
2 Once
3 More than once

Do not read:

7 Don't know / Not sure
9 Refused

10. How often did anyone at least 5 years older than you or an adult try to make you touch them sexually?

(390)

1 Never
2 Once
3 More than once

Do not read:

7 Don't know / Not sure
9 Refused

11. How often did anyone at least 5 years older than you or an adult force you to have sex?

(391)

1 Never
2 Once
3 More than once

Do not read:

7 Don't know / Not sure
9 Refused

As I mentioned when we started this section, I would give you a phone number for an organization that can provide information and referral for these issues. You can dial (place state or local hotline here) to reach a referral service to locate an agency in your area. **[Note: if no local or state hotline is available, give respondent the National Hotline for child abuse 1-800-422-4-A-CHILD (1-800-422-4453).**

Module 23: Random Child Selection

CATI note: If Core Q7.7 = 88, or 99 (No children under age 18 in the household, or Refused), go to next module.

If Core Q7.7 = 1, Interviewer please read: "Previously, you indicated there was one child age 17 or younger in your household. I would like to ask you some questions about that child." **[Go to Q1]**

If Core Q7.7 is >1 and Core Q7.7 does not equal 88 or 99, Interviewer please read: "Previously, you indicated there were **[number]** children age 17 or younger in your household. Think about those **[number]** children in order of their birth, from oldest to youngest. The oldest child is the first child and the youngest child is the last. Please include children with the same birth date, including twins, in the order of their birth."

CATI INSTRUCTION: RANDOMLY SELECT ONE OF THE CHILDREN. This is the "Xth" child. Please substitute "Xth" child's number in all questions below.

INTERVIEWER PLEASE READ:
I have some additional questions about one specific child. The child I will be referring to is the "Xth" **[CATI: please fill in correct number]** child in your household. All following questions about children will be about the "Xth" **[CATI: please fill in]** child.

1. What is the birth month and year of the "**Xth**" child?

(392-397)

_ _ **/**_ _ _ _ Code month and year
7 7/ 7 7 7 7 Don't know / Not sure
9 9/ 9 9 9 9 Refused

CATI INSTRUCTION: Calculate the child's age in months (CHLDAGE1=0 to 216) and also in years (CHLDAGE2=0 to 17) based on the interview date and the birth month and year using a value of 15 for the birth day. If the selected child is < 12 months old enter the calculated months in CHLDAGE1 and 0 in CHLDAGE2. If the child is ≥ 12 months enter the calculated months in CHLDAGE1 and set CHLDAGE2=Truncate (CHLDAGE1/12).

2. Is the child a boy or a girl?

(398)

1 Boy
2 Girl
9 Refused

3. Is the child Hispanic or Latino?

(399)

1 Yes
2 No
7 Don't know / Not sure
9 Refused

4. Which one or more of the following would you say is the race of the child?

(400-405)

[Check all that apply]

Please read:

1 White
2 Black or African American
3 Asian
4 Native Hawaiian or Other Pacific Islander
5 American Indian, Alaska Native

Or

6 Other [specify] ____________________

Do not read:

8 No additional choices
7 Don't know / Not sure
9 Refused

CATI note: If more than one response to Q4, continue. Otherwise, go to Q6.

5. Which one of these groups would you say best represents the child's race? (406)

1 White
2 Black or African American
3 Asian
4 Native Hawaiian or Other Pacific Islander
5 American Indian, Alaska Native
6 Other
7 Don't know / Not sure
9 Refused

6. How are you related to the child? (407)

Please read:

1 Parent (include biologic, step, or adoptive parent)
2 Grandparent
3 Foster parent or guardian
4 Sibling (include biologic, step, and adoptive sibling)
5 Other relative
6 Not related in any way

Do not read:

7 Don't know / Not sure
9 Refused

Module 24: Childhood Asthma Prevalence

CATI note: If response to Core Q7.7 = 88 (None) or 99 (Refused), go to next module.

The next two questions are about the "Xth" **[CATI: please fill in correct number]** child.

1. Has a doctor, nurse or other health professional EVER said that the child has asthma? (408)

1 Yes
2 No **[Go to next module]**
7 Don't know / Not sure **[Go to next module]**
9 Refused **[Go to next module]**

2. Does the child still have asthma? (409)

1 Yes
2 No
7 Don't know / Not sure
9 Refused

Module 25: Childhood Immunization

CATI note: If Core Q7.7 = 88, or 99 (No children under age 18 in the household, or Refused), go to next module.

CATI note: If selected child's age is ≥ 6 months, continue. Otherwise, go to next module.

1. Now I will ask you questions about seasonal flu. There are two types of seasonal flu vaccinations. One is a shot and the other is a spray in the nose. During the past 12 months, has **[Fill: he/she]** had a seasonal flu vaccination?

(410)

1	Yes	
2	No	**[Go to next module]**
7	Don't know / Not sure	**[Go to next module]**
9	Refused	**[Go to next module]**

2. The flu vaccination may have been either the flu shot or the flu spray. The flu spray is the flu vaccination that is sprayed in the nose. During what month and year did **[Fill: he/she]** receive **[Fill: his/her]** most recent seasonal flu vaccination?

(411-416)

_ _ / _ _ _ _	Month / Year
7 7 / 7 7 7 7	Don't know / Not sure
9 9 / 9 9 9 9	Refused

Module 26: HIV/AIDS

CATI NOTE: If Core Q18.1 = 1 (Yes) continue, else go to next module.

1. Where did you have your last HIV test — at a private doctor or HMO office, at a counseling and testing site, at a hospital, at a clinic, in a jail or prison, at a drug treatment facility, at home, or somewhere else?

(417-418)

0 1	Private doctor or HMO office
0 2	Counseling and testing site
0 3	Hospital
0 4	Clinic
0 5	Jail or prison (or other correctional facility)
0 6	Drug treatment facility
0 7	At home
0 8	Somewhere else
7 7	Don't know / Not sure
9 9	Refused

CATI NOTE: If Core Q18.2 = within last 12 months continue, else go to next module.

2. Was it a rapid test where you could get your results within a couple of hours? (419)

1 Yes
2 No
7 Don't know / Not sure
9 Refused

Module 27: Emotional Support and Life Satisfaction

The next two questions are about emotional support and your satisfaction with life.

1. How often do you get the social and emotional support you need?

INTERVIEWER NOTE: If asked, say "please include support from any source." (420)

Please read:

1 Always
2 Usually
3 Sometimes
4 Rarely
5 Never

Do not read:

7 Don't know / Not sure
9 Refused

2. In general, how satisfied are you with your life? (421)

Please read:

1 Very satisfied
2 Satisfied
3 Dissatisfied
4 Very dissatisfied

Do not read:

7 Don't know / Not sure
9 Refused

Asthma Call-Back Permission Script

We would like to call you again within the next 2 weeks to talk in more detail about (your/your child's) experiences with asthma. The information will be used to help develop and improve the asthma programs in <**STATE**>. The information you gave us today and any you give us in the future will be kept confidential. If you agree to this, we will keep your first name or initials and phone number on file, separate from the answers collected today. Even if you agree now, you or others may refuse to participate in the future. Would it be okay if we called you back to ask additional asthma-related questions at a later time?

(422)

1 Yes
2 No

Can I please have either (your/your child's) first name or initials, so we will know who to ask for when we call back?

____________________ Enter first name or initials

Asthma Call-Back Selection

Which person in the household was selected as the focus of the asthma call-back? (423)

1 Adult
2 Child

Can I please have either (your/your child's) first name or initials, so we will know who to ask for when we call back?

____________________ Enter first name or initials

List of Health Problems to Accompany Module 10, Question 3

Lung Problems

- Acute Respiratory Distress Syndrome (ARDS)
- Bronchiectasis
- Bronchopulmonary Dysplasia
- Chronic Obstructive Pulmonary Disease (COPD)
- Cystic Fibrosis
- Emphysema
- Lymphangioleiomyomatosis (LAM)
- Pulmonary Arterial Hypertension
- Sarcoidosis

Kidney Problems

- Chronic Kidney Disease
- Cystitis
- Cystocele (Fallen Bladder)
- Cysts
- Ectopic Kidney
- End-Stage Renal Disease (ESRD)
- Glomerular Diseases
- Interstitial Cystitis
- Kidney Failure
- Kidney Stones
- Nephrotic Syndrome
- Polycystic Kidney Disease
- Pyelonephritis (Kidney Infection)
- Renal Artery Stenosis
- Renal Osteodystrophy
- Renal Tubular Acidosis

Anemia

- Anemia
- Aplastic Anemia
- Fanconi Anemia
- Iron Deficiency Anemia
- Pernicious Anemia
- Sickle Cell Anemia
- Thalassemia

Causes of Weak Immune System

- Cancer
- Chemotherapy
- HIV/AIDS
- Steroids
- Transplant Medicines
- Rheumatoid Arthritis
- Systemic Lupus Erythmatosus (SLE)
